# Approval Disparities for New Drugs in the US and Japan

**DOI:** 10.1001/jamanetworkopen.2025.13640

**Published:** 2025-06-10

**Authors:** Shingo Kasahara, Naohiko Wakutsu, Hiroshi Nakamura

**Affiliations:** 1 Keio University, Yokohama, Japan; 2 Nagoya City University, Nagoya, Aichi, Japan

## Abstract

This cross-sectional study evaluates drug approval disparities between the US and Japan from 2005 to 2022.

## Introduction

The disparity in drug approval between the US and other countries has been widely discussed. Many studies have analyzed approval timing lags for drugs approved in the US and other countries.^1,2,3^ However, an increasing number of drugs approved in the US remain unapproved in other countries, potentially limiting patient access.^4,5,6^ To our knowledge, no statistical analyses of such approval disparities across therapeutic areas have been reported for Japan. This study evaluated drug approval disparities between the US and Japan from 2005 to 2022, focusing on characteristics associated with drugs that remain unapproved.

## Methods

We conducted a cross-sectional analysis of new molecular entities and biologics first approved in the US or Japan between 2005 and 2022. Based on prior research indicating a median approval interval of 1 to 2 years between US and Japanese drug approvals,^2,3^ we assessed each drug’s approval status as of December 31, 2024, allowing a 2-year window for subsequent approval; the drug list is provided in eMethods in Supplement 1. In accordance with the Common Rule, this study was exempt from ethics review and informed consent requirement because no patient data were used. We followed the STROBE reporting guideline.

Data were collected from publicly available databases, including US Food and Drug Administration (FDA) Drugs@FDA, Japanese Pharmaceuticals and Medical Devices Agency, and Kyoto Encyclopedia of Genes and Genomes. Logistic regression was used to identify factors associated with approval disparity among US-approved drugs. The dependent variable was approval disparity dummy, defined as US-approved drugs not approved in Japan by December 31, 2024. The explanatory variables were drug characteristics.

Two-sided *P* < .05 were considered statistically significant. Statistical analyses were performed with SPSS version 29 (IBM). eMethods in Supplement 1 provides additional details.

## Results

Between 2005 and 2022, 711 drugs were approved in the US or Japan. Among 711 drugs, 633 were approved in the US, of which 280 (44.2%) were not approved in Japan (Figure). Conversely, 78 drugs (18.1%) among 431 Japan-approved drugs were not approved in the US. These 78 drugs were considered as local drugs and therefore excluded from the regression analysis because 80.8% (63) of these drugs were not approved by the European Medicines Agency.

**Figure.**
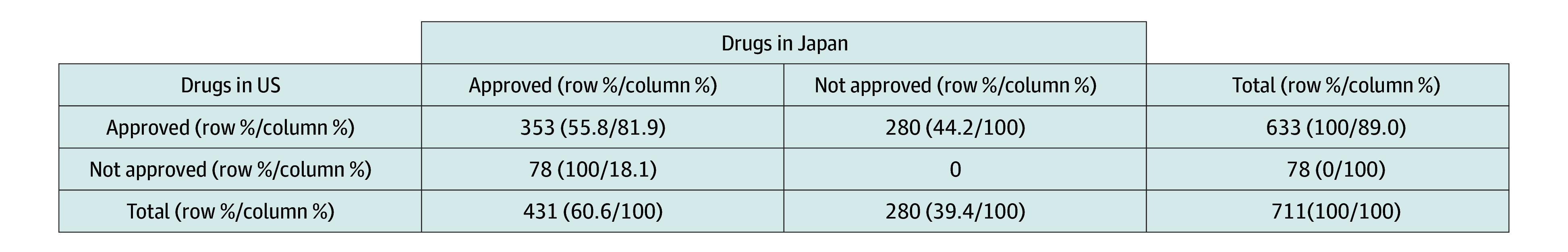
Drugs Approved in the US and Japan From 2005 to 2022 Approval status was determined as of December 31, 2024, allowing a 2-year window for subsequent approvals.

Logistic regression suggested FDA approvals in 2020 to 2022 (β coefficient [SE], 0.95 [0.35]), 2017 to 2019 (β coefficient [SE], 0.90 [0.36]), and 2014 to 2016 (β coefficient [SE], 0.74 [0.37]) showed positive associations with drug disparity dummy (Table). Among the Anatomic Therapeutic Chemical classifications, B (β coefficient [SE], −0.9 [0.4]) and L (β coefficient [SE], −0.93 [0.24]) were inversely associated with approval disparities, indicating a higher likelihood of approval in Japan. Biologics were significantly less likely to remain unapproved (β coefficient [SE], −0.81 [0.23]). None of the 5 expedited FDA review statuses showed associations with approval disparities. Drugs developed by Japanese (β coefficient [SE], −0.87 [0.26]) and global mega (β coefficient [SE], −0.81 [0.23]) pharmaceutical companies were negatively associated with approval disparities. We assessed multicollinearity and found no pairwise correlations exceeding 0.5.

**Table.** Estimated Results of the Logistic Regression Model for Approval Disparities

Independent variable^a^	Estimated β coefficient (SE)	*P* value
FDA approval year		
2020-2022	0.95 (0.35)	.01
2017-2019	0.90 (0.36)	.01
2014-2016	0.74 (0.37)	.045
2011-2013	0.11 (0.38)	.77
2008-2010	1.09 (0.39)	.01
2005-2007	1 [Reference]	
ATC classification		
A: alimentary tract and metabolism	−0.43 (0.31)	.16
B: blood and blood-forming organs	−0.90 (0.40)	.02
J: anti-infective for systemic use	−0.52 (0.31)	.09
L: antineoplastic and immunomodulating agents	−0.93 (0.24)	<.001
N: nervous system	−0.51 (0.31)	.10
Other^b^	1 [Reference]	
Modality		
Biologics	−0.81 (0.23)	<.001
Regulatory status in the US		
Priority review	−0.05 (0.23)	.83
Orphan designation	−0.06 (0.21)	.79
Accelerated approval	−0.19 (0.28)	.49
Fast track designation	0.08 (0.22)	.71
Breakthrough therapy	−0.01 (0.25)	.98
Marketing authorization holder in the US		
Japanese pharmaceutical companies	−0.87 (0.26)	<.001
Global mega pharmaceutical companies	−0.81 (0.23)	<.001

Abbreviations: ATC, Anatomic Therapeutic Chemical; FDA, US Food and Drug Administration.

^a^ Observation coefficient = 633; NagelKerke *R*^2^ = 0.179.

^b^ Other ATC classifications included C: cardiovascular system; D: dermatologicals; G: genitourinary system and sex hormones; H: systemic hormonal preparations, excluding sex hormones and insulins; M: musculoskeletal system; P: antiparasitic products, insecticides, and repellents; R: respiratory system; S: sensory organs; V: various.

## Discussion

Half of US-approved drugs remained unavailable in Japan, highlighting a substantial disparity in available drugs in the US and Japan. In other major pharmaceutical markets, 74.2% of US-approved drugs between 2004 and 2018 were available in Germany,^5^ while 41.3% of US-approved drugs between 2012 and 2019 were available in China.^6^ Both countries lag behind the US, highlighting disparities in global drug access.

Oncology drugs and biologics showed higher approval rates, likely reflecting prioritization in regulatory review due to their clinical importance and market demand. However, a study limitation is that we did not address label expansion, potentially missing approval disparities at the indication level. The findings suggest that optimizing approval pathways to smaller and foreign companies may mitigate approval disparities and improve patient access to innovative treatments in Japan. Addressing these issues could also inform strategies for other countries facing similar challenges.

## Notes

Supplement 1.
eMethods.

eReferences.

Supplement 2.
Data Sharing Statement
