# Supplementary material for: Approval Disparities for New Drugs in the US and Japan
Source: JAMA Netw Open. 2025 Jun 10;8(6):e2513640. doi: 10.1001/jamanetworkopen.2025.13640 (PMC12152698; doi:10.1001/jamanetworkopen.2025.13640)
Supplement: Supplement 1. — eMethods. eReferences. [file jamanetwopen-e2513640-s001.pdf]

Supplemental Online Content

Kasahara S, Wakutsu N, Nakamura H. Approval disparities for new drugs in the US and Japan. *JAMA Netw Open.* 2025;8(6):e2513640. doi:10.1001/jamanetworkopen.2025.13640

eMethods.
eReferences.

This supplemental material has been provided by the authors to give readers additional information about their work.

© 2025 Kasahara S et al. *JAMA Network Open*.

eMethods

We conducted a cross-sectional study of new molecular entities and biologics first approved in the U.S. or Japan between January 1, 2005, and December 31, 2022. We created a cross-tabulation based on whether these drugs were approved in the U.S. and Japan by the end of 2024. Based on prior research indicating a median approval interval of 1–2 years between U.S. and Japanese drug approvals, we assessed each drug's approval status as of December 31, 2024, allowing a 2-year window for subsequent approval.

Furthermore, a regression analysis was conducted, considering the possible influence of confounding factors. The dependent variable, "approval disparity" dummy, was defined as U.S.-approved drugs not approved in Japan as of December 31, 2024 (approval status in Japan at the end of 2024:0,' approved; 1, 'unapproved'), and drug characteristics as explanatory variables to identify factors associated with approval disparities. Statistical analyses were performed using the SPSS version 29.

We used the publicly available Kyoto Encyclopedia of Genes and Genomes (KEGG) Drug Database to identify and list new drugs. The KEGG Drug Database compiles drug approval information for Japan, the U.S., and Europe, including WHO's Anatomic Therapeutic Classification (ATC), modality (new molecular entities or new therapeutic biological products), and MAH names[1]. Additionally, we referred to the Drugs@FDA: FDA-Approved Drugs webpage to verify the information above and supplement the information for U.S. approval dates, MAHs at the time of first approval, and applicability of Expedited Programs for Drug Development (orphan designation, priority review designation, accelerated approval, fast track, and breakthrough therapy) [2,3]. We also used public data from the PMDA

© 2025 Kasahara S et al. *JAMA Network Open*.

to verify drug approval information in Japan and the supplement. To identify global mega pharmaceutical companies, we referred to the top 20 companies listed in the Access to Medicine Index 2022 provided by the Access to Medicine Foundation[4]. The nationality of the MAHs was classified by examining company names and their headquarters' locations.

We also assessed multicollinearity and found no pairwise correlations exceeding 0.5.

This cross-sectional study did not involve clinical data or human participants; therefore, it was not submitted for ethical review. This study adhered to the Strengthening the Reporting of Observational Studies in Epidemiology (STROBE) reporting guidelines.

Drug list

The following table lists the drugs analyzed in this study. Drugs approved in the U.S. are accompanied by their brand names and links to detailed FDA information. Drugs approved only in Japan or lacking Drugs@FDA links have blank fields.

Active ingredient	USA name	FDA Link
Ublituximab	Briumvi	https://www.accessdata.fda.gov/scripts/cder/daf/index.cfm?event=overview.process&ApplNo=761238
Xenon Xe 129 hyperpolarized	Xenoview	https://www.accessdata.fda.gov/scripts/cder/daf/index.cfm?event=overview.process&ApplNo=214375
Nadofaragene firadenovec	Adstiladrin	
Adagrasib	Krazati	https://www.accessdata.fda.gov/scripts/cder/daf/index.cfm?event=overview.process&ApplNo=216340
Olutasidenib	Rezlidhia	https://www.accessdata.fda.gov/scripts/cder/daf/index.cfm?event=overview.process&ApplNo=215814
Etranacogene dezaparvovec	Hemgenix	
Ensitrelvir fumarate		
Teplizumab	Tzield	https://www.accessdata.fda.gov/scripts/cder/daf/index.cfm?event=overview.process&ApplNo=761183
Mirvetuximab soravtansine	Elahere	https://www.accessdata.fda.gov/scripts/cder/daf/index.cfm?event=overview.process&ApplNo=761310

© 2025 Kasahara S et al. *JAMA Network Open*.

Tremelimumab	Imjudo	https://www.accessdata.fda.gov/scripts/cder/daf/index.cfm?event=overview.process&ApplNo=761289
Futibatinib	Lytgobi	https://www.accessdata.fda.gov/scripts/cder/daf/index.cfm?event=overview.process&ApplNo=214801
Valemetostat tosilate		
Ozoralizumab		
Gadopiclenol	Elucirem	https://www.accessdata.fda.gov/scripts/cder/daf/index.cfm?event=overview.process&ApplNo=216986
Terlipressin acetate	Terlivaz	https://www.accessdata.fda.gov/scripts/cder/daf/index.cfm?event=overview.process&ApplNo=022231
Deucravacitinib	Sotyktu	https://www.accessdata.fda.gov/scripts/cder/daf/index.cfm?event=overview.process&ApplNo=214958
Eflapegrastim	Rolvedon	https://www.accessdata.fda.gov/scripts/cder/daf/index.cfm?event=overview.process&ApplNo=761148
Daxibotulinumtoxin A	Daxxify	https://www.accessdata.fda.gov/scripts/cder/daf/index.cfm?event=overview.process&ApplNo=761127
Spesolimab	Spevigo	https://www.accessdata.fda.gov/scripts/cder/daf/index.cfm?event=overview.process&ApplNo=761244
Teclistamab	Tecvayli	https://www.accessdata.fda.gov/scripts/cder/daf/index.cfm?event=overview.process&ApplNo=761291
Lenacapavir sodium	Sunlenca	https://www.accessdata.fda.gov/scripts/cder/daf/index.cfm?event=overview.process&ApplNo=215973 https://www.accessdata.fda.gov/scripts/cder/daf/index.cfm?event=overview.process&ApplNo=215974
Betibeglogene autotemcel	Zynteglo	
Darinaparsin		
Pimitespib		
Vutrisiran sodium	Amvuttra	https://www.accessdata.fda.gov/scripts/cder/daf/index.cfm?event=overview.process&ApplNo=215515
Mosunetuzumab	Lunsumio	https://www.accessdata.fda.gov/scripts/cder/daf/index.cfm?event=overview.process&ApplNo=761263
Tapinarof	Vtama	https://www.accessdata.fda.gov/scripts/cder/daf/index.cfm?event=overview.process&ApplNo=215272
Tirzepatide	Mounjaro	https://www.accessdata.fda.gov/scripts/cder/daf/index.cfm?event=overview.process&ApplNo=215866
Mavacamten	Camzyos	https://www.accessdata.fda.gov/scripts/cder/daf/index.cfm?event=overview.process&ApplNo=214998
Oteseconazole	Vivjoa	https://www.accessdata.fda.gov/scripts/cder/daf/index.cfm?event=overview.process&ApplNo=215888
Olipudase alfa	Xenpozyme	https://www.accessdata.fda.gov/scripts/cder/daf/index.cfm?event=overview.process&ApplNo=761261
Nemolizumab	Nemluvio	https://www.accessdata.fda.gov/scripts/cder/daf/index.cfm?event=overview.process&ApplNo=761390
Ferric derisomaltose		
Carotegrast methyl		
Andexanet alfa		

© 2025 Kasahara S et al. *JAMA Network Open.*

Lutetium Lu 177 vipivotide tetraxetan	Pluvicto	https://www.accessdata.fda.gov/scripts/cder/daf/index.cfm?event=overview.process&ApplNo=215833
Ganaxolone	Ztalmy	https://www.accessdata.fda.gov/scripts/cder/daf/index.cfm?event=overview.process&ApplNo=215904
Pacritinib	Vonjo	https://www.accessdata.fda.gov/scripts/cder/daf/index.cfm?event=overview.process&ApplNo=208712
Ciltacabtagene autoleucel	Carvykti	
Mitapivat sulfate	Pyrukynd	https://www.accessdata.fda.gov/scripts/cder/daf/index.cfm?event=overview.process&ApplNo=216196
Bebtelovimab	Bebtelovimab	
Sutimlimab	Enjaymo	https://www.accessdata.fda.gov/scripts/cder/daf/index.cfm?event=overview.process&ApplNo=761164
Faricimab	Vabysmo	https://www.accessdata.fda.gov/scripts/cder/daf/index.cfm?event=overview.process&ApplNo=761235
Tebentafusp	Kimmtrak	https://www.accessdata.fda.gov/scripts/cder/daf/index.cfm?event=overview.process&ApplNo=761228
Somatrogon	Ngenla	
Gefapixant citrate		
Clazosentan sodium		
Daridorexant hydrochloride	Quviviq	https://www.accessdata.fda.gov/scripts/cder/daf/index.cfm?event=overview.process&ApplNo=214985
Molnupiravir	Molnupiravir	
Nirmatrelvir and ritonavir	Paxlovid	
Inclisiran sodium	Leqvio	https://www.accessdata.fda.gov/scripts/cder/daf/index.cfm?event=overview.process&ApplNo=214012
SARS-CoV-2 Spike glycoprotein vaccine antigen nvx-cov2373		
Tezepelumab	Tezspire	https://www.accessdata.fda.gov/scripts/cder/daf/index.cfm?event=overview.process&ApplNo=761224
Efgartigimod alfa	Vyvgart	https://www.accessdata.fda.gov/scripts/cder/daf/index.cfm?event=overview.process&ApplNo=761195
Tixagevimab and cilgavimab	Evusheld	
Pafolacianine sodium	Cytalux	https://www.accessdata.fda.gov/scripts/cder/daf/index.cfm?event=overview.process&ApplNo=214907
Maribavir	Livtencity	https://www.accessdata.fda.gov/scripts/cder/daf/index.cfm?event=overview.process&ApplNo=215596
Ropeginterferon alfa-2b	Besremi	https://www.accessdata.fda.gov/scripts/cder/daf/index.cfm?event=overview.process&ApplNo=761166

© 2025 Kasahara S et al. *JAMA Network Open.*

Asciminib hydrochloride	Scemblix	https://www.accessdata.fda.gov/scripts/cder/daf/index.cfm?event=overview.process&ApplNo=215358
Maralixibat chloride	Livmarli	
Atogepant	Qulipta	https://www.accessdata.fda.gov/scripts/cder/daf/index.cfm?event=overview.process&ApplNo=215206
Abrocitinib	Cibinqo	https://www.accessdata.fda.gov/scripts/cder/daf/index.cfm?event=overview.process&ApplNo=213871
Avacopan	Tavneos	https://www.accessdata.fda.gov/scripts/cder/daf/index.cfm?event=overview.process&ApplNo=214487
Difamilast		
Tisotumab vedotin	Tivdak	https://www.accessdata.fda.gov/scripts/cder/daf/index.cfm?event=overview.process&ApplNo=761208
Mobocertinib succinate	Exkivity	https://www.accessdata.fda.gov/scripts/cder/daf/index.cfm?event=overview.process&ApplNo=215310
Vosoritide	Voxzogo	https://www.accessdata.fda.gov/scripts/cder/daf/index.cfm?event=overview.process&ApplNo=214938
Lonapegsomatropin	Skytrofa	https://www.accessdata.fda.gov/scripts/cder/daf/index.cfm?event=overview.process&ApplNo=761177
Difelikefalin acetate	Korsuva	https://www.accessdata.fda.gov/scripts/cder/daf/index.cfm?event=overview.process&ApplNo=214916
Bimekizumab	Bimzelx	https://www.accessdata.fda.gov/scripts/cder/daf/index.cfm?event=overview.process&ApplNo=761151
Tick-borne encephalitis vaccine	Ticovac	
Belzutifan	Welireg	https://www.accessdata.fda.gov/scripts/cder/daf/index.cfm?event=overview.process&ApplNo=215383
Avalglucosidase alfa	Nexviazyme	https://www.accessdata.fda.gov/scripts/cder/daf/index.cfm?event=overview.process&ApplNo=761194
Anifrolumab	Saphnelo	https://www.accessdata.fda.gov/scripts/cder/daf/index.cfm?event=overview.process&ApplNo=761123
Elivaldogene autotemcel	Skysona	
Odevixibat	Bylvay	https://www.accessdata.fda.gov/scripts/cder/daf/index.cfm?event=overview.process&ApplNo=215498
Fexinidazole	Fexinidazole	https://www.accessdata.fda.gov/scripts/cder/daf/index.cfm?event=overview.process&ApplNo=214429
Belumosudil mesylate	Rezurock	https://www.accessdata.fda.gov/scripts/cder/daf/index.cfm?event=overview.process&ApplNo=214783
Pneumococcal 15-valent conjugate vaccine	Vaxneuvance	
Finerenone	Kerendia	https://www.accessdata.fda.gov/scripts/cder/daf/index.cfm?event=overview.process&ApplNo=215341
Imeglimin hydrochloride		
Upacicalcet sodium hydrate		
Tucidinostat		

© 2025 Kasahara S et al. *JAMA Network Open*.

Tralokinumab	Adbry	https://www.accessdata.fda.gov/scripts/cder/daf/index.cfm?event=overview.process&ApplNo=761180
Teserpaturev		
Pneumococcal 20-valent conjugate vaccine	Prevnar 20	0
Aducanumab	Aduhelm	https://www.accessdata.fda.gov/scripts/cder/daf/index.cfm?event=overview.process&ApplNo=761178
Plasminogen	Ryplazim	
Ibrexafungerp citrate	Brexafemme	https://www.accessdata.fda.gov/scripts/cder/daf/index.cfm?event=overview.process&ApplNo=214900
Sotorasib	Lumakras	https://www.accessdata.fda.gov/scripts/cder/daf/index.cfm?event=overview.process&ApplNo=214665
Infigratinib phosphate	Truseltiq	https://www.accessdata.fda.gov/scripts/cder/daf/index.cfm?event=overview.process&ApplNo=214622
Sotrovimab	Sotrovimab	
Piflufolastat F-18	Pylarify	https://www.accessdata.fda.gov/scripts/cder/daf/index.cfm?event=overview.process&ApplNo=214793
Amivantamab	Rybrevant	https://www.accessdata.fda.gov/scripts/cder/daf/index.cfm?event=overview.process&ApplNo=761210
Pegcetacoplan	Empaveli	https://www.accessdata.fda.gov/scripts/cder/daf/index.cfm?event=overview.process&ApplNo=215014
Loncastuximab tesirine	Zynlonta	https://www.accessdata.fda.gov/scripts/cder/daf/index.cfm?event=overview.process&ApplNo=761196
Dostarlimab	Jemperli	https://www.accessdata.fda.gov/scripts/cder/daf/index.cfm?event=overview.process&ApplNo=761174
Viloxazine hydrochloride	Qelbree	https://www.accessdata.fda.gov/scripts/cder/daf/index.cfm?event=overview.process&ApplNo=211964
Idecabtagene vicleucel	Abecma	
Pabinafusp alfa		
Fluciclovine F-18		
Dasiglucagon	Zegalogue	https://www.accessdata.fda.gov/scripts/cder/daf/index.cfm?event=overview.process&ApplNo=214231
Ponesimod	Ponvory	https://www.accessdata.fda.gov/scripts/cder/daf/index.cfm?event=overview.process&ApplNo=213498
COVID-19 vaccine (Ad26.COV2-S [recombinant])	Janssen COVID-19 Vaccine	
Fosdenopterin hydrobromide	Nulibry	https://www.accessdata.fda.gov/scripts/cder/daf/index.cfm?event=overview.process&ApplNo=214018

© 2025 Kasahara S et al. *JAMA Network Open.*

Melphalan flufenamide hydrochloride	Pepaxto	https://www.accessdata.fda.gov/scripts/cder/daf/index.cfm?event=overview.process&ApplNo=214383
Casimersen	Amondys 45	https://www.accessdata.fda.gov/scripts/cder/daf/index.cfm?event=overview.process&ApplNo=213026
Trilaciclib dihydrochloride	Cosela	https://www.accessdata.fda.gov/scripts/cder/daf/index.cfm?event=overview.process&ApplNo=214200
Evinacumab	Evkeeza	https://www.accessdata.fda.gov/scripts/cder/daf/index.cfm?event=overview.process&ApplNo=761181
Lisocabtagene maraleucel	Breyanzi	
Umbralisib tosylate	Ukoniq	https://www.accessdata.fda.gov/scripts/cder/daf/index.cfm?event=overview.process&ApplNo=213176
COVID-19 vaccine (ChAdOx1-S [recombinant])		
Voclosporin	Lupkynis	https://www.accessdata.fda.gov/scripts/cder/daf/index.cfm?event=overview.process&ApplNo=213716
Molidustat sodium		
Anamorelin hydrochloride		
Vericiguat	Verquvo	https://www.accessdata.fda.gov/scripts/cder/daf/index.cfm?event=overview.process&ApplNo=214377
Ansuvimab	Ebanga	https://www.accessdata.fda.gov/scripts/cder/daf/index.cfm?event=overview.process&ApplNo=761172
Fenfluramine hydrochloride		https://www.accessdata.fda.gov/scripts/cder/daf/index.cfm?event=overview.process&ApplNo=212102
Elasomeran	Moderna COVID-19 Vaccine	
Rilpivirine		
Cabotegravir sodium	Vocabria	https://www.accessdata.fda.gov/scripts/cder/daf/index.cfm?event=overview.process&ApplNo=212887
Margetuximab	Margenza	https://www.accessdata.fda.gov/scripts/cder/daf/index.cfm?event=overview.process&ApplNo=761150
Tirbanibulin	Klisyri	https://www.accessdata.fda.gov/scripts/cder/daf/index.cfm?event=overview.process&ApplNo=213189
Tozinameran	Pfizer-BioNTech COVID-19 Vaccine, Comirnaty	
Berotralstat hydrochloride	Orladeyo	https://www.accessdata.fda.gov/scripts/cder/daf/index.cfm?event=overview.process&ApplNo=214094

© 2025 Kasahara S et al. *JAMA Network Open.*

Setmelanotide acetate	Imcivree	https://www.accessdata.fda.gov/scripts/cder/daf/index.cfm?event=overview.process&ApplNo=213793
Naxitamab	Danyelza	https://www.accessdata.fda.gov/scripts/cder/daf/index.cfm?event=overview.process&ApplNo=761171
Lonafarnib	Zokinvy	https://www.accessdata.fda.gov/scripts/cder/daf/index.cfm?event=overview.process&ApplNo=213969
Lumasiran sodium	Oxlumo	https://www.accessdata.fda.gov/scripts/cder/daf/index.cfm?event=overview.process&ApplNo=214103
Amikacin sulfate	ARIKAYCE KIT	https://www.accessdata.fda.gov/scripts/cder/daf/index.cfm?event=overview.process&ApplNo=207356
Atoltivimab, maftivimab and odesivimab	Inmazeb	https://www.accessdata.fda.gov/scripts/cder/daf/index.cfm?event=overview.process&ApplNo=761169
Enarodustat		
Sofpironium bromide	Sofdra	https://www.accessdata.fda.gov/scripts/cder/daf/index.cfm?event=overview.process&ApplNo=217347
Filgotinib maleate		
Pralsetinib	Gavreto	https://www.accessdata.fda.gov/scripts/cder/daf/index.cfm?event=overview.process&ApplNo=213721
Copper Cu 64 dotatate	Detectnet	https://www.accessdata.fda.gov/scripts/cder/daf/index.cfm?event=overview.process&ApplNo=213227
Somapacitan	Sogroya	https://www.accessdata.fda.gov/scripts/cder/daf/index.cfm?event=overview.process&ApplNo=761156
Clascoterone	Winlevi	https://www.accessdata.fda.gov/scripts/cder/daf/index.cfm?event=overview.process&ApplNo=213433
Risdiplam	Evrysdi	https://www.accessdata.fda.gov/scripts/cder/daf/index.cfm?event=overview.process&ApplNo=213535
Oliceridine	Olinvyk	https://www.accessdata.fda.gov/scripts/cder/daf/index.cfm?event=overview.process&ApplNo=210730
Nifurtimox	Lampit	https://www.accessdata.fda.gov/scripts/cder/daf/index.cfm?event=overview.process&ApplNo=213464
Belantamab mafodotin	Blenrep	https://www.accessdata.fda.gov/scripts/cder/daf/index.cfm?event=overview.process&ApplNo=761158
Tafasitamab	Monjuvi	https://www.accessdata.fda.gov/scripts/cder/daf/index.cfm?event=overview.process&ApplNo=761163
Brexucabtagene autoleucel	Tecartus	
Abametapir	Xeglyze	https://www.accessdata.fda.gov/scripts/cder/daf/index.cfm?event=overview.process&ApplNo=206966
Fostemsavir tromethamine	Rukobia	https://www.accessdata.fda.gov/scripts/cder/daf/index.cfm?event=overview.process&ApplNo=212950
Triheptanoin	Dojolvi	https://www.accessdata.fda.gov/scripts/cder/daf/index.cfm?event=overview.process&ApplNo=213687
Daprodustat	Jesduvroq	
Satralizumab	Enspryng	https://www.accessdata.fda.gov/scripts/cder/daf/index.cfm?event=overview.process&ApplNo=761149
Vadadustat	Vafseo	https://www.accessdata.fda.gov/scripts/cder/daf/index.cfm?event=overview.process&ApplNo=215192

Lurbinectedin	Zepzelca	https://www.accessdata.fda.gov/scripts/cder/daf/index.cfm?event=overview.process&ApplNo=213702
Inebilizumab	Uplinza	
Flortaucipir F 18	Tauvid	https://www.accessdata.fda.gov/scripts/cder/daf/index.cfm?event=overview.process&ApplNo=212123
Artesunate	Artesunate	
Fluoroestradiol F18	Cerianna	https://www.accessdata.fda.gov/scripts/cder/daf/index.cfm?event=overview.process&ApplNo=212155
Ripretinib	Qinlock	https://www.accessdata.fda.gov/scripts/cder/daf/index.cfm?event=overview.process&ApplNo=213973
Selpercatinib	Retevmo	https://www.accessdata.fda.gov/scripts/cder/daf/index.cfm?event=overview.process&ApplNo=213246
Remdesivir	Veklury	https://www.accessdata.fda.gov/scripts/cder/daf/index.cfm?event=overview.process&ApplNo=214787
Capmatinib hydrochloride	Tabrecta	https://www.accessdata.fda.gov/scripts/cder/daf/index.cfm?event=overview.process&ApplNo=213591
Opicapone	Ongentys	https://www.accessdata.fda.gov/scripts/cder/daf/index.cfm?event=overview.process&ApplNo=212489
Sacituzumab govitecan	Trodelvy	https://www.accessdata.fda.gov/scripts/cder/daf/index.cfm?event=overview.process&ApplNo=761115
Pemigatinib	Pemazyre	https://www.accessdata.fda.gov/scripts/cder/daf/index.cfm?event=overview.process&ApplNo=213736
Tucatinib	Tukysa	https://www.accessdata.fda.gov/scripts/cder/daf/index.cfm?event=overview.process&ApplNo=213411
Selumetinib sulfate	Koselugo	https://www.accessdata.fda.gov/scripts/cder/daf/index.cfm?event=overview.process&ApplNo=213756
Eptacog alfa	Sevenfact	
Tepotinib hydrochloride	Tepmetko	https://www.accessdata.fda.gov/scripts/cder/daf/index.cfm?event=overview.process&ApplNo=214096
Viltolarsen	Viltepso	https://www.accessdata.fda.gov/scripts/cder/daf/index.cfm?event=overview.process&ApplNo=212154
Ozanimod hydrochloride	Zeposia	https://www.accessdata.fda.gov/scripts/cder/daf/index.cfm?event=overview.process&ApplNo=209899
Tirabrutinib hydrochloride		
Isatuximab	Sarclisa	https://www.accessdata.fda.gov/scripts/cder/daf/index.cfm?event=overview.process&ApplNo=761113
Rimegepant sulfate	Nurtec ODT	https://www.accessdata.fda.gov/scripts/cder/daf/index.cfm?event=overview.process&ApplNo=212728
Amisulpride	Barhemsys	https://www.accessdata.fda.gov/scripts/cder/daf/index.cfm?event=overview.process&ApplNo=209510
Eptinezumab	Vyepti	https://www.accessdata.fda.gov/scripts/cder/daf/index.cfm?event=overview.process&ApplNo=761119
Bempedoic acid	Nexletol	https://www.accessdata.fda.gov/scripts/cder/daf/index.cfm?event=overview.process&ApplNo=211616
Tazemetostat hydrobromide	Tazverik	https://www.accessdata.fda.gov/scripts/cder/daf/index.cfm?event=overview.process&ApplNo=211723
Remimazolam besylate	Byfavo	https://www.accessdata.fda.gov/scripts/cder/daf/index.cfm?event=overview.process&ApplNo=212295

© 2025 Kasahara S et al. *JAMA Network Open.*

Delgocitinib		
Dotinurad		
Teprotumumab	Tepezza	https://www.accessdata.fda.gov/scripts/cder/daf/index.cfm?event=overview.process&ApplNo=761143
Avapritinib	Ayvakit	https://www.accessdata.fda.gov/scripts/cder/daf/index.cfm?event=overview.process&ApplNo=212608
Osilodrostat phosphate	Isturisa	https://www.accessdata.fda.gov/scripts/cder/daf/index.cfm?event=overview.process&ApplNo=212801
Purified Vi polysaccharide typhoid vaccine	Typhim Vi	
Ubrogepant	Ubrelvy	https://www.accessdata.fda.gov/scripts/cder/daf/index.cfm?event=overview.process&ApplNo=211765
Trastuzumab deruxtecan	Enhertu	https://www.accessdata.fda.gov/scripts/cder/daf/index.cfm?event=overview.process&ApplNo=761139
Lemborexant	Dayvigo	https://www.accessdata.fda.gov/scripts/cder/daf/index.cfm?event=overview.process&ApplNo=212028
Brilliant blue G	Tissueblue	https://www.accessdata.fda.gov/scripts/cder/daf/index.cfm?event=overview.process&ApplNo=209569
Lumateperone tosylate	Caplyta	https://www.accessdata.fda.gov/scripts/cder/daf/index.cfm?event=overview.process&ApplNo=209500
Enfortumab vedotin	Padcev	https://www.accessdata.fda.gov/scripts/cder/daf/index.cfm?event=overview.process&ApplNo=761137
Golodirsen	Vyondys 53	https://www.accessdata.fda.gov/scripts/cder/daf/index.cfm?event=overview.process&ApplNo=211970
Voxelotor	Oxbryta	https://www.accessdata.fda.gov/scripts/cder/daf/index.cfm?event=overview.process&ApplNo=213137
Cenobamate	Xcopri	https://www.accessdata.fda.gov/scripts/cder/daf/index.cfm?event=overview.process&ApplNo=212839
Givosiran sodium	Givlaari	https://www.accessdata.fda.gov/scripts/cder/daf/index.cfm?event=overview.process&ApplNo=212194
Crizanlizumab	Adakveo	https://www.accessdata.fda.gov/scripts/cder/daf/index.cfm?event=overview.process&ApplNo=761128
Zanubrutinib	Brukinsa	https://www.accessdata.fda.gov/scripts/cder/daf/index.cfm?event=overview.process&ApplNo=213217
Cefiderocol sulfate tosylate	Fetroja	https://www.accessdata.fda.gov/scripts/cder/daf/index.cfm?event=overview.process&ApplNo=209445
Luspatercept	Reblozyl	https://www.accessdata.fda.gov/scripts/cder/daf/index.cfm?event=overview.process&ApplNo=761136
Elexacaftor, ivacaftor and tezacaftor	Trikafta	https://www.accessdata.fda.gov/scripts/cder/daf/index.cfm?event=overview.process&ApplNo=212273
Lasmiditan succinate	Reyvow	https://www.accessdata.fda.gov/scripts/cder/daf/index.cfm?event=overview.process&ApplNo=211280
Fluorodopa F 18	Fluorodopa F 18	https://www.accessdata.fda.gov/scripts/cder/daf/index.cfm?event=overview.process&ApplNo=200655
Afamelanotide acetate	Scenesse	https://www.accessdata.fda.gov/scripts/cder/daf/index.cfm?event=overview.process&ApplNo=210797
Brolucizumab	Beovu	https://www.accessdata.fda.gov/scripts/cder/daf/index.cfm?event=overview.process&ApplNo=761125

© 2025 Kasahara S et al. *JAMA Network Open.*

Trifarotene	Aklief	https://www.accessdata.fda.gov/scripts/cder/daf/index.cfm?event=overview.process&ApplNo=211527
Roxadustat		
Lascufloxacin hydrochloride		
Tenapanor hydrochloride	Ibsrela	https://www.accessdata.fda.gov/scripts/cder/daf/index.cfm?event=overview.process&ApplNo=211801
Edotreotide gallium Ga-68	Ga-68-dotatoc	https://www.accessdata.fda.gov/scripts/cder/daf/index.cfm?event=overview.process&ApplNo=210828
Lefamulin acetate	Xenleta	https://www.accessdata.fda.gov/scripts/cder/daf/index.cfm?event=overview.process&ApplNo=211672
Upadacitinib hydrate	Rinvoq	https://www.accessdata.fda.gov/scripts/cder/daf/index.cfm?event=overview.process&ApplNo=211675
Fedratinib hydrochloride	Inrebic	https://www.accessdata.fda.gov/scripts/cder/daf/index.cfm?event=overview.process&ApplNo=212327
Pretomanid	Pretomanid	https://www.accessdata.fda.gov/scripts/cder/daf/index.cfm?event=overview.process&ApplNo=212862
Pexidartinib hydrochloride	Turalio	https://www.accessdata.fda.gov/scripts/cder/daf/index.cfm?event=overview.process&ApplNo=211810
Darolutamide	Nubeqa	https://www.accessdata.fda.gov/scripts/cder/daf/index.cfm?event=overview.process&ApplNo=212099
Ferric maltol	Accrufer	https://www.accessdata.fda.gov/scripts/cder/daf/index.cfm?event=overview.process&ApplNo=212320
Imipenem, cilastatin and relebactam	Recarbrio	https://www.accessdata.fda.gov/scripts/cder/daf/index.cfm?event=overview.process&ApplNo=212819
Selinexor	Xpovio	https://www.accessdata.fda.gov/scripts/cder/daf/index.cfm?event=overview.process&ApplNo=212306
Bremelanotide	Vyleesi	https://www.accessdata.fda.gov/scripts/cder/daf/index.cfm?event=overview.process&ApplNo=210557
Entrectinib	Rozlytrek	https://www.accessdata.fda.gov/scripts/cder/daf/index.cfm?event=overview.process&ApplNo=212725
Quizartinib dihydrochloride	Vanflyta	https://www.accessdata.fda.gov/scripts/cder/daf/index.cfm?event=overview.process&ApplNo=216993
Polatuzumab vedotin	Polivy	https://www.accessdata.fda.gov/scripts/cder/daf/index.cfm?event=overview.process&ApplNo=761121
Alpelisib	Piqray	https://www.accessdata.fda.gov/scripts/cder/daf/index.cfm?event=overview.process&ApplNo=212526
Onasemnogene abeparvovec	Zolgensma	
Erdafitinib	Balversa	https://www.accessdata.fda.gov/scripts/cder/daf/index.cfm?event=overview.process&ApplNo=212018
Siponimod fumarate	Mayzent	https://www.accessdata.fda.gov/scripts/cder/daf/index.cfm?event=overview.process&ApplNo=209884
Risankizumab	Skyrizi	https://www.accessdata.fda.gov/scripts/cder/daf/index.cfm?event=overview.process&ApplNo=761105

© 2025 Kasahara S et al. *JAMA Network Open.*

Beperminogene perplasmid		
Sodium selenite		
Peficitinib hydrobromide		
Solriamfetol hydrochloride	Sunosi	https://www.accessdata.fda.gov/scripts/cder/daf/index.cfm?event=overview.process&ApplNo=211230
Brexanolone	Zulresso	https://www.accessdata.fda.gov/scripts/cder/daf/index.cfm?event=overview.process&ApplNo=211371
Triclabendazole	Egaten	https://www.accessdata.fda.gov/scripts/cder/daf/index.cfm?event=overview.process&ApplNo=208711
Prabotulinumtoxin A	Jeuvea	https://www.accessdata.fda.gov/scripts/cder/daf/index.cfm?event=overview.process&ApplNo=761085
Relugolix	Orgovyx	https://www.accessdata.fda.gov/scripts/cder/daf/index.cfm?event=overview.process&ApplNo=214621
Romosozumab	Evenity	https://www.accessdata.fda.gov/scripts/cder/daf/index.cfm?event=overview.process&ApplNo=761062
Esaxerenone		
Mirogabalin besylate		
Tagraxofusp	Elzonris	https://www.accessdata.fda.gov/scripts/cder/daf/index.cfm?event=overview.process&ApplNo=761116
Ravulizumab	Ultomiris	https://www.accessdata.fda.gov/scripts/cder/daf/index.cfm?event=overview.process&ApplNo=761108
Calaspargase pegol	Asparlas	https://www.accessdata.fda.gov/scripts/cder/daf/index.cfm?event=overview.process&ApplNo=761102
Prucalopride succinate	Motegrity	https://www.accessdata.fda.gov/scripts/cder/daf/index.cfm?event=overview.process&ApplNo=210166
Larotrectinib sulfate	Vitrakvi	https://www.accessdata.fda.gov/scripts/cder/daf/index.cfm?event=overview.process&ApplNo=210861
Glasdegib maleate	Daurismo	https://www.accessdata.fda.gov/scripts/cder/daf/index.cfm?event=overview.process&ApplNo=210656
Emapalumab	Gamifant	https://www.accessdata.fda.gov/scripts/cder/daf/index.cfm?event=overview.process&ApplNo=761107
Rifamycin sodium	Aemcolo	https://www.accessdata.fda.gov/scripts/cder/daf/index.cfm?event=overview.process&ApplNo=210910
Revefenacin	Yupelri	https://www.accessdata.fda.gov/scripts/cder/daf/index.cfm?event=overview.process&ApplNo=210598
Talazoparib tosylate	Talzenna	https://www.accessdata.fda.gov/scripts/cder/daf/index.cfm?event=overview.process&ApplNo=211651
Elapegademase	Revcovi	https://www.accessdata.fda.gov/scripts/cder/daf/index.cfm?event=overview.process&ApplNo=761092
Omadacycline tosylate	Nuzyra	https://www.accessdata.fda.gov/scripts/cder/daf/index.cfm?event=overview.process&ApplNo=209816
Sarecycline	Seysara	https://www.accessdata.fda.gov/scripts/cder/daf/index.cfm?event=overview.process&ApplNo=209521
Cemiplimab	Libtayo	https://www.accessdata.fda.gov/scripts/cder/daf/index.cfm?event=overview.process&ApplNo=761097

© 2025 Kasahara S et al. *JAMA Network Open*.

Dacomitinib	Vizimpro	https://www.accessdata.fda.gov/scripts/cder/daf/index.cfm?event=overview.process&ApplNo=211288
Galcanezumab	Emgality	https://www.accessdata.fda.gov/scripts/cder/daf/index.cfm?event=overview.process&ApplNo=761063
Duvelisib hydrate	Copiktra	https://www.accessdata.fda.gov/scripts/cder/daf/index.cfm?event=overview.process&ApplNo=211155
Omidenepag isopropyl	Omlonti	https://www.accessdata.fda.gov/scripts/cder/daf/index.cfm?event=overview.process&ApplNo=215092
Vibegron	Gemtesa	https://www.accessdata.fda.gov/scripts/cder/daf/index.cfm?event=overview.process&ApplNo=213006
Gilteritinib fumarate	Xospata	https://www.accessdata.fda.gov/scripts/cder/daf/index.cfm?event=overview.process&ApplNo=211349
Lorlatinib	Lorbrena	https://www.accessdata.fda.gov/scripts/cder/daf/index.cfm?event=overview.process&ApplNo=210868
Fremanezumab	Ajovy	https://www.accessdata.fda.gov/scripts/cder/daf/index.cfm?event=overview.process&ApplNo=761089
Moxetumomab pasudotox	Lumoxiti	https://www.accessdata.fda.gov/scripts/cder/daf/index.cfm?event=overview.process&ApplNo=761104
Caplacizumab	Cablivi	https://www.accessdata.fda.gov/scripts/cder/daf/index.cfm?event=overview.process&ApplNo=761112
Doravirine	Pifeltro	https://www.accessdata.fda.gov/scripts/cder/daf/index.cfm?event=overview.process&ApplNo=210806
Lanadelumab	Takhzyro	https://www.accessdata.fda.gov/scripts/cder/daf/index.cfm?event=overview.process&ApplNo=761090
Patisiran sodium	Onpattro	https://www.accessdata.fda.gov/scripts/cder/daf/index.cfm?event=overview.process&ApplNo=210922
Elagolix sodium	Orilissa	https://www.accessdata.fda.gov/scripts/cder/daf/index.cfm?event=overview.process&ApplNo=210450
Ivosidenib	Tibsovo	https://www.accessdata.fda.gov/scripts/cder/daf/index.cfm?event=overview.process&ApplNo=211192
Tafenoquine succinate	Krintafel	https://www.accessdata.fda.gov/scripts/cder/daf/index.cfm?event=overview.process&ApplNo=210795
Tecovirimat hydrate	TPOXX	https://www.accessdata.fda.gov/scripts/cder/daf/index.cfm?event=overview.process&ApplNo=208627
Inotersen sodium	Tegsedi	https://www.accessdata.fda.gov/scripts/cder/daf/index.cfm?event=overview.process&ApplNo=211172
Binimetinib	Mektovi	https://www.accessdata.fda.gov/scripts/cder/daf/index.cfm?event=overview.process&ApplNo=210498
Encorafenib	Braftovi	https://www.accessdata.fda.gov/scripts/cder/daf/index.cfm?event=overview.process&ApplNo=210496
Cannabidiol	Epidiolex	https://www.accessdata.fda.gov/scripts/cder/daf/index.cfm?event=overview.process&ApplNo=210365
Plazomicin sulfate	Zemdri	https://www.accessdata.fda.gov/scripts/cder/daf/index.cfm?event=overview.process&ApplNo=210303
Pegvaliase	Palynziq	https://www.accessdata.fda.gov/scripts/cder/daf/index.cfm?event=overview.process&ApplNo=761079
Avatrombopag maleate	Doptelet	https://www.accessdata.fda.gov/scripts/cder/daf/index.cfm?event=overview.process&ApplNo=210238
Erenumab	Aimovig	https://www.accessdata.fda.gov/scripts/cder/daf/index.cfm?event=overview.process&ApplNo=761077
Fostamatinib disodium	Tavalisse	https://www.accessdata.fda.gov/scripts/cder/daf/index.cfm?event=overview.process&ApplNo=209299

© 2025 Kasahara S et al. *JAMA Network Open*.

Darvadstrocel		0
Condoliase		0
Evocalcet		0
Sodium zirconium cyclosilicate	Lokelma	https://www.accessdata.fda.gov/scripts/cder/daf/index.cfm?event=overview.process&ApplNo=207078
Tildrakizumab	Ilumya	https://www.accessdata.fda.gov/scripts/cder/daf/index.cfm?event=overview.process&ApplNo=761067
Ibalizumab	Trogarzo	https://www.accessdata.fda.gov/scripts/cder/daf/index.cfm?event=overview.process&ApplNo=761065
Baloxavir marboxil	Xofluza	https://www.accessdata.fda.gov/scripts/cder/daf/index.cfm?event=overview.process&ApplNo=210854
Burosumab	Crysvita	https://www.accessdata.fda.gov/scripts/cder/daf/index.cfm?event=overview.process&ApplNo=761068
Apalutamide	Erleada	https://www.accessdata.fda.gov/scripts/cder/daf/index.cfm?event=overview.process&ApplNo=210951
Tezacaftor and ivacaftor	Symdeko	https://www.accessdata.fda.gov/scripts/cder/daf/index.cfm?event=overview.process&ApplNo=210491
Bictegravir, emtricitabine and tenofovir alafenamide	Biktarvy	https://www.accessdata.fda.gov/scripts/cder/daf/index.cfm?event=overview.process&ApplNo=210251
Lutetium (177Lu) oxodotreotide	Lutathera	https://www.accessdata.fda.gov/scripts/cder/daf/index.cfm?event=overview.process&ApplNo=208700
Fosravuconazole L-lysine ethanolate		
Elobixibat hydrate		
Angiotensin II	Giapreza	https://www.accessdata.fda.gov/scripts/cder/daf/index.cfm?event=overview.process&ApplNo=209360
Macimorelin acetate	Macrilen	https://www.accessdata.fda.gov/scripts/cder/daf/index.cfm?event=overview.process&ApplNo=205598
Ertugliflozin pidolate	Steglatro	https://www.accessdata.fda.gov/scripts/cder/daf/index.cfm?event=overview.process&ApplNo=209803
Voretigene neparvovec	Luxturna	
Netarsudil mesylate	Rhopressa	https://www.accessdata.fda.gov/scripts/cder/daf/index.cfm?event=overview.process&ApplNo=208254
Semaglutide	Ozempic	https://www.accessdata.fda.gov/scripts/cder/daf/index.cfm?event=overview.process&ApplNo=209637
Emicizumab	Hemlibra	https://www.accessdata.fda.gov/scripts/cder/daf/index.cfm?event=overview.process&ApplNo=761083
Vestronidase alfa	Mepsevii	https://www.accessdata.fda.gov/scripts/cder/daf/index.cfm?event=overview.process&ApplNo=761047
Benralizumab	Fasenra	https://www.accessdata.fda.gov/scripts/cder/daf/index.cfm?event=overview.process&ApplNo=761070
Letermovir	Prevymis	https://www.accessdata.fda.gov/scripts/cder/daf/index.cfm?event=overview.process&ApplNo=209939

© 2025 Kasahara S et al. *JAMA Network Open*.

Latanoproste ne bunod	Vyzulta	https://www.accessdata.fda.gov/scripts/cder/daf/index.cfm?event=overview.process&ApplNo=207795
Acalabrutinib	Calquenc e	https://www.accessdata.fda.gov/scripts/cder/daf/index.cfm?event=overview.process&ApplNo=210259
Axicabtagene ciloleucel	Yescarta	
Abemaciclib	Verzenio	https://www.accessdata.fda.gov/scripts/cder/daf/index.cfm?event=overview.process&ApplNo=208716
Rupatadine fumarate		
Secnidazole	Solosec	https://www.accessdata.fda.gov/scripts/cder/daf/index.cfm?event=overview.process&ApplNo=209363
Copanlisib	Aliqopa	https://www.accessdata.fda.gov/scripts/cder/daf/index.cfm?event=overview.process&ApplNo=209936
Tisagenlecleu cel-T	Kymriah	
Meropenem and vaborbactam	Vabomer e	https://www.accessdata.fda.gov/scripts/cder/daf/index.cfm?event=overview.process&ApplNo=209776
Benznidazole	Benznida zole	https://www.accessdata.fda.gov/scripts/cder/daf/index.cfm?event=overview.process&ApplNo=209570
Tivozanib hydrochloride	Fotivda	https://www.accessdata.fda.gov/scripts/cder/daf/index.cfm?event=overview.process&ApplNo=212904
Enasidenib mesylate	Idhifa	https://www.accessdata.fda.gov/scripts/cder/daf/index.cfm?event=overview.process&ApplNo=209606
Glecaprevir and pibrentasvir	Mavyret	https://www.accessdata.fda.gov/scripts/cder/daf/index.cfm?event=overview.process&ApplNo=209394
Sofosbuvir, velpatasvir and voxilaprevir	Vosevi	https://www.accessdata.fda.gov/scripts/cder/daf/index.cfm?event=overview.process&ApplNo=209195
Neratinib maleate	Nerlynx	https://www.accessdata.fda.gov/scripts/cder/daf/index.cfm?event=overview.process&ApplNo=208051
Guselkumab	Tremfya	https://www.accessdata.fda.gov/scripts/cder/daf/index.cfm?event=overview.process&ApplNo=761061
Cenegermin	Oxervate	
Amenamevir		
Pemafibrate		
Inotuzumab ozogamicin	Besponsa	https://www.accessdata.fda.gov/scripts/cder/daf/index.cfm?event=overview.process&ApplNo=761040
Betrixaban	Bevyxxa	https://www.accessdata.fda.gov/scripts/cder/daf/index.cfm?event=overview.process&ApplNo=208383
Delafloxacin meglumine	Baxdela	https://www.accessdata.fda.gov/scripts/cder/daf/index.cfm?event=overview.process&ApplNo=208610
Sarilumab	Kevzara	https://www.accessdata.fda.gov/scripts/cder/daf/index.cfm?event=overview.process&ApplNo=761037
Durvalumab	Imfinzi	https://www.accessdata.fda.gov/scripts/cder/daf/index.cfm?event=overview.process&ApplNo=761069
Brigatinib	Alunbrig	https://www.accessdata.fda.gov/scripts/cder/daf/index.cfm?event=overview.process&ApplNo=208772

© 2025 Kasahara S et al. *JAMA Network Open.*

Midostaurin	Rydapt	https://www.accessdata.fda.gov/scripts/cder/daf/index.cfm?event=overview.process&ApplNo=207997
Abaloparatide	Tymlos	https://www.accessdata.fda.gov/scripts/cder/daf/index.cfm?event=overview.process&ApplNo=208743
Cerliponase alfa	Brineura	https://www.accessdata.fda.gov/scripts/cder/daf/index.cfm?event=overview.process&ApplNo=761052
Valbenazine tosylate	Ingrezza	https://www.accessdata.fda.gov/scripts/cder/daf/index.cfm?event=overview.process&ApplNo=209241
Deutetrabenazine	Austedo	https://www.accessdata.fda.gov/scripts/cder/daf/index.cfm?event=overview.process&ApplNo=208082
Forodesine hydrochloride		
Ocrelizumab	Ocrevus	https://www.accessdata.fda.gov/scripts/cder/daf/index.cfm?event=overview.process&ApplNo=761053
Dupilumab	Dupixent	https://www.accessdata.fda.gov/scripts/cder/daf/index.cfm?event=overview.process&ApplNo=761055
Niraparib tosilate hydrate	Zejula	https://www.accessdata.fda.gov/scripts/cder/daf/index.cfm?event=overview.process&ApplNo=208447
Avelumab	Bavencio	https://www.accessdata.fda.gov/scripts/cder/daf/index.cfm?event=overview.process&ApplNo=761049
Naldemedine tosylate	Symproic	https://www.accessdata.fda.gov/scripts/cder/daf/index.cfm?event=overview.process&ApplNo=208854
Ribociclib succinate	Kisqali	https://www.accessdata.fda.gov/scripts/cder/daf/index.cfm?event=overview.process&ApplNo=209092
Telotristat ethyl	Xermelo	https://www.accessdata.fda.gov/scripts/cder/daf/index.cfm?event=overview.process&ApplNo=208794
Baricitinib	Olumiant	https://www.accessdata.fda.gov/scripts/cder/daf/index.cfm?event=overview.process&ApplNo=207924
Deflazacort	Emflaza	https://www.accessdata.fda.gov/scripts/cder/daf/index.cfm?event=overview.process&ApplNo=208684
Plecanatide	Trulance	https://www.accessdata.fda.gov/scripts/cder/daf/index.cfm?event=overview.process&ApplNo=208745
Nusinersen sodium	Spinraza	https://www.accessdata.fda.gov/scripts/cder/daf/index.cfm?event=overview.process&ApplNo=209531
Crisantaspase		
Rucaparib camsylate	Rubraca	https://www.accessdata.fda.gov/scripts/cder/daf/index.cfm?event=overview.process&ApplNo=209115
Crisaborole	Eucrisa	https://www.accessdata.fda.gov/scripts/cder/daf/index.cfm?event=overview.process&ApplNo=207695
Etelcalcetide hydrochloride	Parsavib	
Bezlotoxumab	Zinplava	https://www.accessdata.fda.gov/scripts/cder/daf/index.cfm?event=overview.process&ApplNo=761046
Olaratumab	Lartruvo	https://www.accessdata.fda.gov/scripts/cder/daf/index.cfm?event=overview.process&ApplNo=761038
Bilastine		
Eteplirsen	Exondys 51	https://www.accessdata.fda.gov/scripts/cder/daf/index.cfm?event=overview.process&ApplNo=206448
Lifitegrast	Xiidra	https://www.accessdata.fda.gov/scripts/cder/daf/index.cfm?event=overview.process&ApplNo=208073

© 2025 Kasahara S et al. *JAMA Network Open*.

Brodalumab	Siliq	https://www.accessdata.fda.gov/scripts/cder/daf/index.cfm?event=overview.process&ApplNo=761032
Sofosbuvir and velpatasvir	Epclusa	https://www.accessdata.fda.gov/scripts/cder/daf/index.cfm?event=overview.process&ApplNo=208341
Obeticholic acid	Ocaliva	https://www.accessdata.fda.gov/scripts/cder/daf/index.cfm?event=overview.process&ApplNo=207999
Daclizumab	Zinbryta	https://www.accessdata.fda.gov/scripts/cder/daf/index.cfm?event=overview.process&ApplNo=761029
Migalastat hydrochloride	Galafold	https://www.accessdata.fda.gov/scripts/cder/daf/index.cfm?event=overview.process&ApplNo=208623
Atezolizumab	Tecentriq	https://www.accessdata.fda.gov/scripts/cder/daf/index.cfm?event=overview.process&ApplNo=761034
Pimavanserin tartrate	Nuplazid	https://www.accessdata.fda.gov/scripts/cder/daf/index.cfm?event=overview.process&ApplNo=207318
Venetoclax	Venclexta	https://www.accessdata.fda.gov/scripts/cder/daf/index.cfm?event=overview.process&ApplNo=208573
Reslizumab	Cinqair	https://www.accessdata.fda.gov/scripts/cder/daf/index.cfm?event=overview.process&ApplNo=761033
Ixekizumab	Taltz	https://www.accessdata.fda.gov/scripts/cder/daf/index.cfm?event=overview.process&ApplNo=125521
Obiltoxaximab	Anthim	https://www.accessdata.fda.gov/scripts/cder/daf/index.cfm?event=overview.process&ApplNo=125509
Elbasvir and grazoprevir	Zepatier	https://www.accessdata.fda.gov/scripts/cder/daf/index.cfm?event=overview.process&ApplNo=208261
Brivaracetam	Briviact	https://www.accessdata.fda.gov/scripts/cder/daf/index.cfm?event=overview.process&ApplNo=205836
Selexipag	Uptravi	https://www.accessdata.fda.gov/scripts/cder/daf/index.cfm?event=overview.process&ApplNo=207947
Lesinurad	Zurampic	https://www.accessdata.fda.gov/scripts/cder/daf/index.cfm?event=overview.process&ApplNo=207988
Elotuzumab	Empliciti	https://www.accessdata.fda.gov/scripts/cder/daf/index.cfm?event=overview.process&ApplNo=761035
Necitumumab	Portrazza	https://www.accessdata.fda.gov/scripts/cder/daf/index.cfm?event=overview.process&ApplNo=125547
Ixazomib citrate	Ninlaro	https://www.accessdata.fda.gov/scripts/cder/daf/index.cfm?event=overview.process&ApplNo=208462
Daratumumab	Darzalex	https://www.accessdata.fda.gov/scripts/cder/daf/index.cfm?event=overview.process&ApplNo=761036
Osimertinib mesylate	Tagrisso	https://www.accessdata.fda.gov/scripts/cder/daf/index.cfm?event=overview.process&ApplNo=208065
Cobimetinib fumarate	Cotellic	https://www.accessdata.fda.gov/scripts/cder/daf/index.cfm?event=overview.process&ApplNo=206192
Elvitegravir, cobicistat, emtricitabine and tenofovir alafenamide	Genvoya	https://www.accessdata.fda.gov/scripts/cder/daf/index.cfm?event=overview.process&ApplNo=207561
Mepolizumab	Nucala	https://www.accessdata.fda.gov/scripts/cder/daf/index.cfm?event=overview.process&ApplNo=125526
Talimogene laherparepvec	Imlygic	

© 2025 Kasahara S et al. *JAMA Network Open*.

Patiromer sorbitex calcium	Veltassa	https://www.accessdata.fda.gov/scripts/cder/daf/index.cfm?event=overview.process&ApplNo=205739
Idarucizumab	Praxbind	https://www.accessdata.fda.gov/scripts/cder/daf/index.cfm?event=overview.process&ApplNo=761025
Aripiprazole lauroxil	Aristada	https://www.accessdata.fda.gov/scripts/cder/daf/index.cfm?event=overview.process&ApplNo=207533
Lusutrombopag	Mulpleta	https://www.accessdata.fda.gov/scripts/cder/daf/index.cfm?event=overview.process&ApplNo=210923
Ozenoxacin	Xepi	https://www.accessdata.fda.gov/scripts/cder/daf/index.cfm?event=overview.process&ApplNo=208945
Cariprazine hydrochloride	Vraylar	https://www.accessdata.fda.gov/scripts/cder/daf/index.cfm?event=overview.process&ApplNo=204370
Uridine triacetate	Xuriden	https://www.accessdata.fda.gov/scripts/cder/daf/index.cfm?event=overview.process&ApplNo=208169
Rolapitant hydrochloride	Varubi	https://www.accessdata.fda.gov/scripts/cder/daf/index.cfm?event=overview.process&ApplNo=206500
Sebelipase alfa	Kanuma	https://www.accessdata.fda.gov/scripts/cder/daf/index.cfm?event=overview.process&ApplNo=125561
Flibanserin	Addyi	https://www.accessdata.fda.gov/scripts/cder/daf/index.cfm?event=overview.process&ApplNo=022526
Alirocumab	Praluent	https://www.accessdata.fda.gov/scripts/cder/daf/index.cfm?event=overview.process&ApplNo=125559
Sonidegib phosphate	Odomzo	https://www.accessdata.fda.gov/scripts/cder/daf/index.cfm?event=overview.process&ApplNo=205266
Evolocumab	Repatha	https://www.accessdata.fda.gov/scripts/cder/daf/index.cfm?event=overview.process&ApplNo=125522
Brexpiprazole	Rexulti	https://www.accessdata.fda.gov/scripts/cder/daf/index.cfm?event=overview.process&ApplNo=205422
Sacubitril valsartan sodium hydrate	Entresto	https://www.accessdata.fda.gov/scripts/cder/daf/index.cfm?event=overview.process&ApplNo=207620
Asfotase alfa	Strensiq	https://www.accessdata.fda.gov/scripts/cder/daf/index.cfm?event=overview.process&ApplNo=125513
Antithrombin gamma		
Olanexidine gluconate		
Lumacaftor and ivacaftor	Orkambi	https://www.accessdata.fda.gov/scripts/cder/daf/index.cfm?event=overview.process&ApplNo=206038
Eluxadoline		https://www.accessdata.fda.gov/scripts/cder/daf/index.cfm?event=overview.process&ApplNo=206940
Deoxycholic acid	Kybella	https://www.accessdata.fda.gov/scripts/cder/daf/index.cfm?event=overview.process&ApplNo=206333
Trelagliptin succinate		
Cangrelor	Kengreal	https://www.accessdata.fda.gov/scripts/cder/daf/index.cfm?event=overview.process&ApplNo=204958
Dinutuximab	Unituxin	https://www.accessdata.fda.gov/scripts/cder/daf/index.cfm?event=overview.process&ApplNo=125516

© 2025 Kasahara S et al. *JAMA Network Open.*

Isavuconazonium sulfate	Cresemba	https://www.accessdata.fda.gov/scripts/cder/daf/index.cfm?event=overview.process&ApplNo=207500
Ceftazidime and avibactam	Avycaz	https://www.accessdata.fda.gov/scripts/cder/daf/index.cfm?event=overview.process&ApplNo=206494
Safinamide mesylate	Xadago	https://www.accessdata.fda.gov/scripts/cder/daf/index.cfm?event=overview.process&ApplNo=207145
Panobinostat lactate	Farydak	https://www.accessdata.fda.gov/scripts/cder/daf/index.cfm?event=overview.process&ApplNo=205353
Lenvatinib mesylate	Lenvima	https://www.accessdata.fda.gov/scripts/cder/daf/index.cfm?event=overview.process&ApplNo=206947
Palbociclib	Ibrance	https://www.accessdata.fda.gov/scripts/cder/daf/index.cfm?event=overview.process&ApplNo=207103
Parathyroid hormone (human)	Natpara	https://www.accessdata.fda.gov/scripts/cder/daf/index.cfm?event=overview.process&ApplNo=125511
Secukinumab	Cosentyx	https://www.accessdata.fda.gov/scripts/cder/daf/index.cfm?event=overview.process&ApplNo=125504
Vonoprazan fumarate		
Ceftolozane sulfate and tazobactam sodium	Zerbaxa	https://www.accessdata.fda.gov/scripts/cder/daf/index.cfm?event=overview.process&ApplNo=206829
Dasabuvir, ombitasvir, paritaprevir and ritonavir	Viekira Pak	https://www.accessdata.fda.gov/scripts/cder/daf/index.cfm?event=overview.process&ApplNo=206619
Finafloxacin	Xtoro	https://www.accessdata.fda.gov/scripts/cder/daf/index.cfm?event=overview.process&ApplNo=206307
Olaparib	Lynparza	https://www.accessdata.fda.gov/scripts/cder/daf/index.cfm?event=overview.process&ApplNo=206162
Blinatumomab	Blincyto	https://www.accessdata.fda.gov/scripts/cder/daf/index.cfm?event=overview.process&ApplNo=125557
Nintedanib esylate	Ofev	https://www.accessdata.fda.gov/scripts/cder/daf/index.cfm?event=overview.process&ApplNo=205832
Fosnetupitant and palonosetron hydrochloride	Akynzeo	https://www.accessdata.fda.gov/scripts/cder/daf/index.cfm?event=overview.process&ApplNo=205718
Ledipasvir and sofosbuvir	Harvoni	https://www.accessdata.fda.gov/scripts/cder/daf/index.cfm?event=overview.process&ApplNo=205834
Dulaglutide	Trulicity	https://www.accessdata.fda.gov/scripts/cder/daf/index.cfm?event=overview.process&ApplNo=125469
Naloxegol oxalate	Movantik	https://www.accessdata.fda.gov/scripts/cder/daf/index.cfm?event=overview.process&ApplNo=204760
Pembrolizumab	Keytruda	https://www.accessdata.fda.gov/scripts/cder/daf/index.cfm?event=overview.process&ApplNo=125514
Eliglustat tartrate	Cerdelga	https://www.accessdata.fda.gov/scripts/cder/daf/index.cfm?event=overview.process&ApplNo=205494

© 2025 Kasahara S et al. *JAMA Network Open.*

Suvorexant	Belsomra	https://www.accessdata.fda.gov/scripts/cder/daf/index.cfm?event=overview.process&ApplNo=204569
Oritavancin diphosphate	Orbactiv	https://www.accessdata.fda.gov/scripts/cder/daf/index.cfm?event=overview.process&ApplNo=206334
Olodaterol hydrochloride	Striverdi Respimat	https://www.accessdata.fda.gov/scripts/cder/daf/index.cfm?event=overview.process&ApplNo=203108
Idelalisib	Zydelig	https://www.accessdata.fda.gov/scripts/cder/daf/index.cfm?event=overview.process&ApplNo=205858
Peginterferon beta-1a	Plegridy	https://www.accessdata.fda.gov/scripts/cder/daf/index.cfm?event=overview.process&ApplNo=125499
Tavaborole	Kerydin	https://www.accessdata.fda.gov/scripts/cder/daf/index.cfm?event=overview.process&ApplNo=204427
Alectinib hydrochloride	Alecensa	https://www.accessdata.fda.gov/scripts/cder/daf/index.cfm?event=overview.process&ApplNo=208434
Nivolumab	Opdivo	https://www.accessdata.fda.gov/scripts/cder/daf/index.cfm?event=overview.process&ApplNo=125554
Daclatasvir dihydrochloride	Daklinza	https://www.accessdata.fda.gov/scripts/cder/daf/index.cfm?event=overview.process&ApplNo=206843
Belinostat	Beleodaq	https://www.accessdata.fda.gov/scripts/cder/daf/index.cfm?event=overview.process&ApplNo=206256
Tedizolid phosphate	Sivextro	https://www.accessdata.fda.gov/scripts/cder/daf/index.cfm?event=overview.process&ApplNo=205435
Efinaconazole	Jublia	https://www.accessdata.fda.gov/scripts/cder/daf/index.cfm?event=overview.process&ApplNo=203567
Dalbavancin	Dalvance	https://www.accessdata.fda.gov/scripts/cder/daf/index.cfm?event=overview.process&ApplNo=021883
Empagliflozin	Jardiance	https://www.accessdata.fda.gov/scripts/cder/daf/index.cfm?event=overview.process&ApplNo=204629
Vedolizumab	Entyvio	https://www.accessdata.fda.gov/scripts/cder/daf/index.cfm?event=overview.process&ApplNo=125476
Vorapaxar sulfate	Zontivity	https://www.accessdata.fda.gov/scripts/cder/daf/index.cfm?event=overview.process&ApplNo=204886
Ceritinib	Zykadia	https://www.accessdata.fda.gov/scripts/cder/daf/index.cfm?event=overview.process&ApplNo=205755
Delamanid		
Siltuximab	Sylvant	https://www.accessdata.fda.gov/scripts/cder/daf/index.cfm?event=overview.process&ApplNo=125496
Ramucirumab	Cyramza	https://www.accessdata.fda.gov/scripts/cder/daf/index.cfm?event=overview.process&ApplNo=125477
Trifluridine and tipiracil hydrochloride	Lonsurf	https://www.accessdata.fda.gov/scripts/cder/daf/index.cfm?event=overview.process&ApplNo=207981
Luseogliflozin hydrate		
Favipiravir		
Tofogliflozin		
Apremilast	Otezla	https://www.accessdata.fda.gov/scripts/cder/daf/index.cfm?event=overview.process&ApplNo=205437
Albiglutide	Tanzeum	https://www.accessdata.fda.gov/scripts/cder/daf/index.cfm?event=overview.process&ApplNo=125431

© 2025 Kasahara S et al. *JAMA Network Open*.

Miltefosine	Impavido	https://www.accessdata.fda.gov/scripts/cder/daf/index.cfm?event=overview.process&ApplNo=204684
Florbetaben (18F)	Neuraceq	https://www.accessdata.fda.gov/scripts/cder/daf/index.cfm?event=overview.process&ApplNo=204677
Droxidopa	Northera	https://www.accessdata.fda.gov/scripts/cder/daf/index.cfm?event=overview.process&ApplNo=203202
Elosulfase alfa	Vimizim	https://www.accessdata.fda.gov/scripts/cder/daf/index.cfm?event=overview.process&ApplNo=125460
Tasimelteon	Hetlioz	https://www.accessdata.fda.gov/scripts/cder/daf/index.cfm?event=overview.process&ApplNo=205677
Ipragliflozin L-proline		
Umeclidinium bromide and vilanterol	Anoro Ellipta	https://www.accessdata.fda.gov/scripts/cder/daf/index.cfm?event=overview.process&ApplNo=203975
Sofosbuvir	Sovaldi	https://www.accessdata.fda.gov/scripts/cder/daf/index.cfm?event=overview.process&ApplNo=204671
Simeprevir sodium	Olysio	https://www.accessdata.fda.gov/scripts/cder/daf/index.cfm?event=overview.process&ApplNo=205123
Ibrutinib	Imbruvica	https://www.accessdata.fda.gov/scripts/cder/daf/index.cfm?event=overview.process&ApplNo=205552
Obinutuzumab	Gazyva	https://www.accessdata.fda.gov/scripts/cder/daf/index.cfm?event=overview.process&ApplNo=125486
Flutemetamol F 18	Vizamyl	https://www.accessdata.fda.gov/scripts/cder/daf/index.cfm?event=overview.process&ApplNo=203137
Defibrotide sodium	Defitelio	https://www.accessdata.fda.gov/scripts/cder/daf/index.cfm?event=overview.process&ApplNo=208114
Macitentan	Opsumit	https://www.accessdata.fda.gov/scripts/cder/daf/index.cfm?event=overview.process&ApplNo=204410
Riociguat	Adempas	https://www.accessdata.fda.gov/scripts/cder/daf/index.cfm?event=overview.process&ApplNo=204819
Conjugated estrogens and bazedoxifene acetate	Duavee	https://www.accessdata.fda.gov/scripts/cder/daf/index.cfm?event=overview.process&ApplNo=022247
Vortioxetine hydrobromide	Brintellix	
Tafamidis meglumine	Vyndaqel	https://www.accessdata.fda.gov/scripts/cder/daf/index.cfm?event=overview.process&ApplNo=211996
Cholic acid	Cholbam	https://www.accessdata.fda.gov/scripts/cder/daf/index.cfm?event=overview.process&ApplNo=205750
Dolutegravir sodium	Tivicay	https://www.accessdata.fda.gov/scripts/cder/daf/index.cfm?event=overview.process&ApplNo=204790
Afatinib dimaleate	Gilotrif	https://www.accessdata.fda.gov/scripts/cder/daf/index.cfm?event=overview.process&ApplNo=201292
Topiroxostat		
Trametinib dimethyl sulfoxide	Mekinist	https://www.accessdata.fda.gov/scripts/cder/daf/index.cfm?event=overview.process&ApplNo=204114
Dabrafenib mesylate	Tafinlar	https://www.accessdata.fda.gov/scripts/cder/daf/index.cfm?event=overview.process&ApplNo=202806

© 2025 Kasahara S et al. *JAMA Network Open.*

Radium Ra 223 dichloride	Xofigo	https://www.accessdata.fda.gov/scripts/cder/daf/index.cfm?event=overview.process&ApplNo=203971
Fluticasone furoate and vilanterol trifenatate	Breo Ellipta	https://www.accessdata.fda.gov/scripts/cder/daf/index.cfm?event=overview.process&ApplNo=204275
Canagliflozin	Invokana	https://www.accessdata.fda.gov/scripts/cder/daf/index.cfm?event=overview.process&ApplNo=204042
Dimethyl fumarate	Tecfidera	https://www.accessdata.fda.gov/scripts/cder/daf/index.cfm?event=overview.process&ApplNo=204063
Istradefylline	Nourianz	https://www.accessdata.fda.gov/scripts/cder/daf/index.cfm?event=overview.process&ApplNo=022075
Metreleptin	Myalept	https://www.accessdata.fda.gov/scripts/cder/daf/index.cfm?event=overview.process&ApplNo=125390
Acotiamide hydrochloride		
Ospemifene	Osphena	https://www.accessdata.fda.gov/scripts/cder/daf/index.cfm?event=overview.process&ApplNo=203505
Trastuzumab emtansine	Kadcyla	https://www.accessdata.fda.gov/scripts/cder/daf/index.cfm?event=overview.process&ApplNo=125427
Pomalidomide	Pomalyst	https://www.accessdata.fda.gov/scripts/cder/daf/index.cfm?event=overview.process&ApplNo=204026
Lixisenatide	Adlyxin	https://www.accessdata.fda.gov/scripts/cder/daf/index.cfm?event=overview.process&ApplNo=208471
Mipomersen sodium	Kynamro	https://www.accessdata.fda.gov/scripts/cder/daf/index.cfm?event=overview.process&ApplNo=203568
Crofelemer	Fulyzaq	
Bedaquiline fumarate	Sirturo	https://www.accessdata.fda.gov/scripts/cder/daf/index.cfm?event=overview.process&ApplNo=204384
Lomitapide mesylate	Juxtapid	https://www.accessdata.fda.gov/scripts/cder/daf/index.cfm?event=overview.process&ApplNo=203858
Anacaulase	NexoBrid	https://www.accessdata.fda.gov/scripts/cder/daf/index.cfm?event=overview.process&ApplNo=761192
Ponatinib hydrochloride	Iclusig	https://www.accessdata.fda.gov/scripts/cder/daf/index.cfm?event=overview.process&ApplNo=203469
Raxibacumab	raxibacumab	https://www.accessdata.fda.gov/scripts/cder/daf/index.cfm?event=overview.process&ApplNo=125349
Cabozantinib s-malate	Cometriq	https://www.accessdata.fda.gov/scripts/cder/daf/index.cfm?event=overview.process&ApplNo=203756
Dapagliflozin propanediol	Farxiga	https://www.accessdata.fda.gov/scripts/cder/daf/index.cfm?event=overview.process&ApplNo=202293
Tofacitinib citrate	Xeljanz	https://www.accessdata.fda.gov/scripts/cder/daf/index.cfm?event=overview.process&ApplNo=203214
Omacetaxine mepesuccinate	Synribo	https://www.accessdata.fda.gov/scripts/cder/daf/index.cfm?event=overview.process&ApplNo=203585
Ocriplasmin	Jetrea	https://www.accessdata.fda.gov/scripts/cder/daf/index.cfm?event=overview.process&ApplNo=125422
Insulin degludec	Tresiba	https://www.accessdata.fda.gov/scripts/cder/daf/index.cfm?event=overview.process&ApplNo=203314
Anagliptin		

© 2025 Kasahara S et al. *JAMA Network Open*.

Regorafenib hydrate	Stivarga	https://www.accessdata.fda.gov/scripts/cder/daf/index.cfm?event=overview.process&ApplNo=203085
Teriflunomide	Aubagio	https://www.accessdata.fda.gov/scripts/cder/daf/index.cfm?event=overview.process&ApplNo=202992
Choline C-11	Choline C 11 Injection	https://www.accessdata.fda.gov/scripts/cder/daf/index.cfm?event=overview.process&ApplNo=203155
Bosutinib hydrate	Bosulif	https://www.accessdata.fda.gov/scripts/cder/daf/index.cfm?event=overview.process&ApplNo=203341
Enzalutamide	Xtandi	https://www.accessdata.fda.gov/scripts/cder/daf/index.cfm?event=overview.process&ApplNo=203415
Teduglutide	Gattex	https://www.accessdata.fda.gov/scripts/cder/daf/index.cfm?event=overview.process&ApplNo=203441
Linaclotide	Linzess	https://www.accessdata.fda.gov/scripts/cder/daf/index.cfm?event=overview.process&ApplNo=202811
Elvitegravir, cobicistat, emtricitabine and tenofovir disoproxil	Stribild	https://www.accessdata.fda.gov/scripts/cder/daf/index.cfm?event=overview.process&ApplNo=203100
Aflibercept	Zaltrap	https://www.accessdata.fda.gov/scripts/cder/daf/index.cfm?event=overview.process&ApplNo=125418
Perampanel hydrate	Fycompa	https://www.accessdata.fda.gov/scripts/cder/daf/index.cfm?event=overview.process&ApplNo=202834
Carfilzomib	Kyprolis	https://www.accessdata.fda.gov/scripts/cder/daf/index.cfm?event=overview.process&ApplNo=202714
Aclidinium bromide	Tudorza Pressair	https://www.accessdata.fda.gov/scripts/cder/daf/index.cfm?event=overview.process&ApplNo=202450
Iguratimod		
Teneligliptin hydrobromide hydrate		
Lorcaserin hydrochloride	Belviq	
Pertuzumab	Perjeta	https://www.accessdata.fda.gov/scripts/cder/daf/index.cfm?event=overview.process&ApplNo=125409
Taliglucerase alfa	Elelyso	https://www.accessdata.fda.gov/scripts/cder/daf/index.cfm?event=overview.process&ApplNo=022458
Avanafil	Stendra	https://www.accessdata.fda.gov/scripts/cder/daf/index.cfm?event=overview.process&ApplNo=202276
Pasireotide diaspartate	Signifor	https://www.accessdata.fda.gov/scripts/cder/daf/index.cfm?event=overview.process&ApplNo=200677
Florbetapir F-18	Amyvid	https://www.accessdata.fda.gov/scripts/cder/daf/index.cfm?event=overview.process&ApplNo=202008
Mogamulizumab	Poteligeo	https://www.accessdata.fda.gov/scripts/cder/daf/index.cfm?event=overview.process&ApplNo=761051
Peginesatide acetate	Omontys	https://www.accessdata.fda.gov/scripts/cder/daf/index.cfm?event=overview.process&ApplNo=202799
Lucinactant	Surfaxin	https://www.accessdata.fda.gov/scripts/cder/daf/index.cfm?event=overview.process&ApplNo=021746
Ivacaftor	Kalydeco	https://www.accessdata.fda.gov/scripts/cder/daf/index.cfm?event=overview.process&ApplNo=203188

© 2025 Kasahara S et al. *JAMA Network Open.*

Vismodegib	Erivedge	https://www.accessdata.fda.gov/scripts/cder/daf/index.cfm?event=overview.process&ApplNo=203388
Axitinib	Inlyta	https://www.accessdata.fda.gov/scripts/cder/daf/index.cfm?event=overview.process&ApplNo=202324
Ingenol mebutate	Picato	https://www.accessdata.fda.gov/scripts/cder/daf/index.cfm?event=overview.process&ApplNo=202833
Azilsartan		
Glucarpidase	Voraxaze	https://www.accessdata.fda.gov/scripts/cder/daf/index.cfm?event=overview.process&ApplNo=125327
Asparaginase	Erwinaze	
Aflibercept	Zaltrap	https://www.accessdata.fda.gov/scripts/cder/daf/index.cfm?event=overview.process&ApplNo=125418
Ruxolitinib phosphate	Jakafi	https://www.accessdata.fda.gov/scripts/cder/daf/index.cfm?event=overview.process&ApplNo=202192
Crizotinib	Xalkori	https://www.accessdata.fda.gov/scripts/cder/daf/index.cfm?event=overview.process&ApplNo=202570
Brentuximab vedotin	Adcetris	https://www.accessdata.fda.gov/scripts/cder/daf/index.cfm?event=overview.process&ApplNo=125388
Vemurafenib	Zelboraf	https://www.accessdata.fda.gov/scripts/cder/daf/index.cfm?event=overview.process&ApplNo=202429
Mirabegron	Myrbetriq	https://www.accessdata.fda.gov/scripts/cder/daf/index.cfm?event=overview.process&ApplNo=202611
Azficel-T	Laviv	
Belatacept	Nulojix	https://www.accessdata.fda.gov/scripts/cder/daf/index.cfm?event=overview.process&ApplNo=125288
Fidaxomicin	Dificid	https://www.accessdata.fda.gov/scripts/cder/daf/index.cfm?event=overview.process&ApplNo=201699
Telaprevir	Incivek	https://www.accessdata.fda.gov/scripts/cder/daf/index.cfm?event=overview.process&ApplNo=201917
Rilpivirine hydrochloride	Edurant	https://www.accessdata.fda.gov/scripts/cder/daf/index.cfm?event=overview.process&ApplNo=202022
Apixaban	Eliquis	https://www.accessdata.fda.gov/scripts/cder/daf/index.cfm?event=overview.process&ApplNo=202155
Boceprevir	Victrelis	https://www.accessdata.fda.gov/scripts/cder/daf/index.cfm?event=overview.process&ApplNo=202258
Linagliptin	Tradjenta	https://www.accessdata.fda.gov/scripts/cder/daf/index.cfm?event=overview.process&ApplNo=201280
Abiraterone acetate	Zytiga	https://www.accessdata.fda.gov/scripts/cder/daf/index.cfm?event=overview.process&ApplNo=202379
Edoxaban tosilate hydrate	Savaysa	https://www.accessdata.fda.gov/scripts/cder/daf/index.cfm?event=overview.process&ApplNo=206316
Vandetanib	Caprelsa	https://www.accessdata.fda.gov/scripts/cder/daf/index.cfm?event=overview.process&ApplNo=022405
Gabapentin enacarbil	Horizant	https://www.accessdata.fda.gov/scripts/cder/daf/index.cfm?event=overview.process&ApplNo=022399
Ezogabine	Potiga	https://www.accessdata.fda.gov/scripts/cder/daf/index.cfm?event=overview.process&ApplNo=022345
Ipilimumab	Yervoy	https://www.accessdata.fda.gov/scripts/cder/daf/index.cfm?event=overview.process&ApplNo=125377

© 2025 Kasahara S et al. *JAMA Network Open*.

Gadobutrol	Gadavist	https://www.accessdata.fda.gov/scripts/cder/daf/index.cfm?event=overview.process&ApplNo=201277
Belimumab	Benlysta	https://www.accessdata.fda.gov/scripts/cder/daf/index.cfm?event=overview.process&ApplNo=125370
Azilsartan kamedoxomil	Edarbi	https://www.accessdata.fda.gov/scripts/cder/daf/index.cfm?event=overview.process&ApplNo=200796
Eldecalcitol		
Vilazodone hydrochloride	Viibryd	https://www.accessdata.fda.gov/scripts/cder/daf/index.cfm?event=overview.process&ApplNo=022567
Spinosad	Natroba	https://www.accessdata.fda.gov/scripts/cder/daf/index.cfm?event=overview.process&ApplNo=022408
Ticagrelor	Brilinta	https://www.accessdata.fda.gov/scripts/cder/daf/index.cfm?event=overview.process&ApplNo=022433
Eribulin mesylate	Eribulin mesylate	https://www.accessdata.fda.gov/scripts/cder/daf/index.cfm?event=overview.process&ApplNo=201532
Tesamorelin acetate	Egrifta	https://www.accessdata.fda.gov/scripts/cder/daf/index.cfm?event=overview.process&ApplNo=022505
Ceftaroline fosamil	Ceftaroline fosamil for injection	https://www.accessdata.fda.gov/scripts/cder/daf/index.cfm?event=overview.process&ApplNo=200327
Lurasidone hydrochloride	Lurasidone HCl	https://www.accessdata.fda.gov/scripts/cder/daf/index.cfm?event=overview.process&ApplNo=200603
Fingolimod hydrochloride	Fingolimod HCl oral capsules	
Pegloticase	Krystexxa	https://www.accessdata.fda.gov/scripts/cder/daf/index.cfm?event=overview.process&ApplNo=125293
Laninamivir octanoate hydrate		
Incobotulinumtoxin A	Xeomin	https://www.accessdata.fda.gov/scripts/cder/daf/index.cfm?event=overview.process&ApplNo=125360
Alcaftadine	Lastacaft	https://www.accessdata.fda.gov/scripts/cder/daf/index.cfm?event=overview.process&ApplNo=022134
Roflumilast	Daliresp	https://www.accessdata.fda.gov/scripts/cder/daf/index.cfm?event=overview.process&ApplNo=022522
Cabazitaxel acetonate	Jevtana	https://www.accessdata.fda.gov/scripts/cder/daf/index.cfm?event=overview.process&ApplNo=201023
Denosumab	Prolia	https://www.accessdata.fda.gov/scripts/cder/daf/index.cfm?event=overview.process&ApplNo=125320
Dienogest and estradiol valerate	Natazia	https://www.accessdata.fda.gov/scripts/cder/daf/index.cfm?event=overview.process&ApplNo=022252
Sipuleucel-T	Provenge, Provenge	
Alogliptin benzoate	Nesina	https://www.accessdata.fda.gov/scripts/cder/daf/index.cfm?event=overview.process&ApplNo=022271

© 2025 Kasahara S et al. *JAMA Network Open.*

Polidocanol	Asclera	https://www.accessdata.fda.gov/scripts/cder/daf/index.cfm?event=overview.process&ApplNo=021201
Velaglucerase alfa	Vpriv	https://www.accessdata.fda.gov/scripts/cder/daf/index.cfm?event=overview.process&ApplNo=022575
Collagenase clostridium histolyticum	Xiaflex	https://www.accessdata.fda.gov/scripts/cder/daf/index.cfm?event=overview.process&ApplNo=125338
Dalfampridine	Ampyra	https://www.accessdata.fda.gov/scripts/cder/daf/index.cfm?event=overview.process&ApplNo=022250
Peramivir	Rapivab	https://www.accessdata.fda.gov/scripts/cder/daf/index.cfm?event=overview.process&ApplNo=206426
Ecallantide	Kalbitor	https://www.accessdata.fda.gov/scripts/cder/daf/index.cfm?event=overview.process&ApplNo=125277
Indacaterol maleate	Arcapta Neohaler	https://www.accessdata.fda.gov/scripts/cder/daf/index.cfm?event=overview.process&ApplNo=022383
Romidepsin	Romidepsin for infusion	https://www.accessdata.fda.gov/scripts/cder/daf/index.cfm?event=overview.process&ApplNo=022393
Ofatumumab	Arzerra	https://www.accessdata.fda.gov/scripts/cder/daf/index.cfm?event=overview.process&ApplNo=125326
Pazopanib hydrochloride	Votrient tablets	https://www.accessdata.fda.gov/scripts/cder/daf/index.cfm?event=overview.process&ApplNo=022465
Miriplatin hydrate		
Pralatrexate	Folotyn	https://www.accessdata.fda.gov/scripts/cder/daf/index.cfm?event=overview.process&ApplNo=022468
Telavancin hydrochloride	Telavancin	https://www.accessdata.fda.gov/scripts/cder/daf/index.cfm?event=overview.process&ApplNo=022110
Vigabatrin	Sabril (Vigabatrin) tablet 500mg	https://www.accessdata.fda.gov/scripts/cder/daf/index.cfm?event=overview.process&ApplNo=020427
Asenapine maleate	Saphris	https://www.accessdata.fda.gov/scripts/cder/daf/index.cfm?event=overview.process&ApplNo=022117
Saxagliptin hydrochloride	Onglyza	https://www.accessdata.fda.gov/scripts/cder/daf/index.cfm?event=overview.process&ApplNo=022350
Dronedarone hydrochloride	Multaq	https://www.accessdata.fda.gov/scripts/cder/daf/index.cfm?event=overview.process&ApplNo=022425
Liraglutide	Victoza	https://www.accessdata.fda.gov/scripts/cder/daf/index.cfm?event=overview.process&ApplNo=022341
Canakinumab	Ilaris	https://www.accessdata.fda.gov/scripts/cder/daf/index.cfm?event=overview.process&ApplNo=125319
Besifloxacin hydrochloride	Besifloxacin HCl	https://www.accessdata.fda.gov/scripts/cder/daf/index.cfm?event=overview.process&ApplNo=022308
Tolvaptan	Samsca	https://www.accessdata.fda.gov/scripts/cder/daf/index.cfm?event=overview.process&ApplNo=022275
Ulipristal acetate	Ella	https://www.accessdata.fda.gov/scripts/cder/daf/index.cfm?event=overview.process&ApplNo=022474
Capsaicin	Qutenza	https://www.accessdata.fda.gov/scripts/cder/daf/index.cfm?event=overview.process&ApplNo=022395
Iloperidone	Fanapt	https://www.accessdata.fda.gov/scripts/cder/daf/index.cfm?event=overview.process&ApplNo=022192

© 2025 Kasahara S et al. *JAMA Network Open.*

Abobotulinu mtoxin A	Dysport	https://www.accessdata.fda.gov/scripts/cder/daf/index.cfm?event=overview.process&ApplNo=125274
Golimumab	Simponi	https://www.accessdata.fda.gov/scripts/cder/daf/index.cfm?event=overview.process&ApplNo=125289
Eslicarbazepi ne acetate	Aptiom	https://www.accessdata.fda.gov/scripts/cder/daf/index.cfm?event=overview.process&ApplNo=022416
Bazedoxifene acetate		
Benzyl alcohol	Ulesfia	https://www.accessdata.fda.gov/scripts/cder/daf/index.cfm?event=overview.process&ApplNo=022129
Artemether and lumefantrine	Coartem	https://www.accessdata.fda.gov/scripts/cder/daf/index.cfm?event=overview.process&ApplNo=022268
Prasugrel hydrochloride	Effient	https://www.accessdata.fda.gov/scripts/cder/daf/index.cfm?event=overview.process&ApplNo=022307
Nalfurafine hydrochloride		
Minodronic acid hydrate		
Ustekinumab	Stelara	https://www.accessdata.fda.gov/scripts/cder/daf/index.cfm?event=overview.process&ApplNo=125261
Milnacipran hydrochloride	Savella tablets	https://www.accessdata.fda.gov/scripts/cder/daf/index.cfm?event=overview.process&ApplNo=022256
Degarelix acetate	Degareli x	https://www.accessdata.fda.gov/scripts/cder/daf/index.cfm?event=overview.process&ApplNo=022201
Gadofosveset trisodium	Vasovist	
Plerixafor	Mozobil	https://www.accessdata.fda.gov/scripts/cder/daf/index.cfm?event=overview.process&ApplNo=022311
Fospropofol disodium	Lusedra	https://www.accessdata.fda.gov/scripts/cder/daf/index.cfm?event=overview.process&ApplNo=022244
Eltrombopag olamine	Promacta	https://www.accessdata.fda.gov/scripts/cder/daf/index.cfm?event=overview.process&ApplNo=022291
Tapentadol hydrochloride	Tapentad ol	https://www.accessdata.fda.gov/scripts/cder/daf/index.cfm?event=overview.process&ApplNo=022304
Tafluprost	Zioptan	https://www.accessdata.fda.gov/scripts/cder/daf/index.cfm?event=overview.process&ApplNo=202514
Pirfenidone	Esbriet	https://www.accessdata.fda.gov/scripts/cder/daf/index.cfm?event=overview.process&ApplNo=022535
Rivaroxaban	Xarelto	https://www.accessdata.fda.gov/scripts/cder/daf/index.cfm?event=overview.process&ApplNo=022406
Iobenguane sulfate I 123	AdreVie w	https://www.accessdata.fda.gov/scripts/cder/daf/index.cfm?event=overview.process&ApplNo=022290
Filgrastim	Neutrova l	
Lacosamide	Vimpat rablets	https://www.accessdata.fda.gov/scripts/cder/daf/index.cfm?event=overview.process&ApplNo=022253
Romiplostim	Nplate	https://www.accessdata.fda.gov/scripts/cder/daf/index.cfm?event=overview.process&ApplNo=125268
Tetrabenazin e	Xenazine	https://www.accessdata.fda.gov/scripts/cder/daf/index.cfm?event=overview.process&ApplNo=021894

© 2025 Kasahara S et al. *JAMA Network Open.*

Clevidipine	Cleviprex	https://www.accessdata.fda.gov/scripts/cder/daf/index.cfm?event=overview.process&ApplNo=022156
Sugammadex sodium	Bridion	https://www.accessdata.fda.gov/scripts/cder/daf/index.cfm?event=overview.process&ApplNo=022225
Icatibant acetate	Firazyr	https://www.accessdata.fda.gov/scripts/cder/daf/index.cfm?event=overview.process&ApplNo=022150
Difluprednate	Durezol	https://www.accessdata.fda.gov/scripts/cder/daf/index.cfm?event=overview.process&ApplNo=022212
Alvimopan	Entereg	https://www.accessdata.fda.gov/scripts/cder/daf/index.cfm?event=overview.process&ApplNo=021775
Methylnaltrexone bromide	Relistor	https://www.accessdata.fda.gov/scripts/cder/daf/index.cfm?event=overview.process&ApplNo=021964
Certolizumab pegol	Cimzia	https://www.accessdata.fda.gov/scripts/cder/daf/index.cfm?event=overview.process&ApplNo=125160
Febuxostat	Uloric (Febuxostat) tablets	https://www.accessdata.fda.gov/scripts/cder/daf/index.cfm?event=overview.process&ApplNo=021856
Regadenoson	Lexiscan	https://www.accessdata.fda.gov/scripts/cder/daf/index.cfm?event=overview.process&ApplNo=022161
Bendamustine hydrochloride	Treanda	https://www.accessdata.fda.gov/scripts/cder/daf/index.cfm?event=overview.process&ApplNo=022249
Dabigatran etexilate mesylate	Pradaxa	https://www.accessdata.fda.gov/scripts/cder/daf/index.cfm?event=overview.process&ApplNo=022512
Desvenlafaxine succinate	Pristiq	https://www.accessdata.fda.gov/scripts/cder/daf/index.cfm?event=overview.process&ApplNo=021992
Rilonacept	Arcalyst	https://www.accessdata.fda.gov/scripts/cder/daf/index.cfm?event=overview.process&ApplNo=125249
Sitafloxacin		
Blonanserin		
Thrombomodulin alfa		
Etravirine	Intelence	https://www.accessdata.fda.gov/scripts/cder/daf/index.cfm?event=overview.process&ApplNo=022187
Fosaprepitant dimeglumine		
Nebivolol hydrochloride	Bystolic	https://www.accessdata.fda.gov/scripts/cder/daf/index.cfm?event=overview.process&ApplNo=021742
Sapropterin dihydrochloride	Kuvan	https://www.accessdata.fda.gov/scripts/cder/daf/index.cfm?event=overview.process&ApplNo=022181
Nilotinib hydrochloride hydrate	Tasigna	https://www.accessdata.fda.gov/scripts/cder/daf/index.cfm?event=overview.process&ApplNo=022068
Gadoxetate disodium	Eovist injection	https://www.accessdata.fda.gov/scripts/cder/daf/index.cfm?event=overview.process&ApplNo=022090
Ixabepilone	Ixempra	https://www.accessdata.fda.gov/scripts/cder/daf/index.cfm?event=overview.process&ApplNo=022065
Raltegravir potassium	Isentress	https://www.accessdata.fda.gov/scripts/cder/daf/index.cfm?event=overview.process&ApplNo=022145

© 2025 Kasahara S et al. *JAMA Network Open*.

Trabectedin	Yondelis	https://www.accessdata.fda.gov/scripts/cder/daf/index.cfm?event=overview.process&ApplNo=207953
Lanreotide acetate	Somatuline Depot	https://www.accessdata.fda.gov/scripts/cder/daf/index.cfm?event=overview.process&ApplNo=022074
Ammonia N 13	Ammonia N13	https://www.accessdata.fda.gov/scripts/cder/daf/index.cfm?event=overview.process&ApplNo=022119
Maraviroc	Selzentry	https://www.accessdata.fda.gov/scripts/cder/daf/index.cfm?event=overview.process&ApplNo=022128
Garenoxacin mesylate		
Epoetin beta pegol	Mircera	https://www.accessdata.fda.gov/scripts/cder/daf/index.cfm?event=overview.process&ApplNo=125164
Ambrisentan	Letairis	https://www.accessdata.fda.gov/scripts/cder/daf/index.cfm?event=overview.process&ApplNo=022081
Temsirolimus	Torisel	https://www.accessdata.fda.gov/scripts/cder/daf/index.cfm?event=overview.process&ApplNo=022088
Fesoterodine fumarate	Toviaz	https://www.accessdata.fda.gov/scripts/cder/daf/index.cfm?event=overview.process&ApplNo=022030
Imidafenacin		
Retapamulin	Altabax	https://www.accessdata.fda.gov/scripts/cder/daf/index.cfm?event=overview.process&ApplNo=022055
Eculizumab	Soliris	https://www.accessdata.fda.gov/scripts/cder/daf/index.cfm?event=overview.process&ApplNo=125166
Lapatinib ditosylate	Tykerb	https://www.accessdata.fda.gov/scripts/cder/daf/index.cfm?event=overview.process&ApplNo=022059
Aliskiren fumarate	Tekturna	https://www.accessdata.fda.gov/scripts/cder/daf/index.cfm?event=overview.process&ApplNo=021985
Lisdexamfetamine dimesylate	Vyvanse	https://www.accessdata.fda.gov/scripts/cder/daf/index.cfm?event=overview.process&ApplNo=021977
Betaine		
Everolimus	Afinitor	https://www.accessdata.fda.gov/scripts/cder/daf/index.cfm?event=overview.process&ApplNo=022334
Rufinamide	Banzel oral tabs	https://www.accessdata.fda.gov/scripts/cder/daf/index.cfm?event=overview.process&ApplNo=021911
Stiripentol	Diacomit	https://www.accessdata.fda.gov/scripts/cder/daf/index.cfm?event=overview.process&ApplNo=206709
Paliperidone	Invega	https://www.accessdata.fda.gov/scripts/cder/daf/index.cfm?event=overview.process&ApplNo=021999
Sinecatechins	Veregen	https://www.accessdata.fda.gov/scripts/cder/daf/index.cfm?event=overview.process&ApplNo=021902
Telbivudine	Tyzeka	https://www.accessdata.fda.gov/scripts/cder/daf/index.cfm?event=overview.process&ApplNo=022011
Ciclesonide	Omnaris	https://www.accessdata.fda.gov/scripts/cder/daf/index.cfm?event=overview.process&ApplNo=022004
Sitagliptin phosphate	Januvia	https://www.accessdata.fda.gov/scripts/cder/daf/index.cfm?event=overview.process&ApplNo=021995
Vorinostat	Zolinza	https://www.accessdata.fda.gov/scripts/cder/daf/index.cfm?event=overview.process&ApplNo=021991
Bismuth subcitrate, tetracycline	Pylera	https://www.accessdata.fda.gov/scripts/cder/daf/index.cfm?event=overview.process&ApplNo=050786

© 2025 Kasahara S et al. *JAMA Network Open.*

and metronidazole		
Panitumumab	Vectibix	https://www.accessdata.fda.gov/scripts/cder/daf/index.cfm?event=overview.process&ApplNo=125147
Mozavaptane hydrochloride		
Idursulfase	Elaprase	https://www.accessdata.fda.gov/scripts/cder/daf/index.cfm?event=overview.process&ApplNo=125151
Ranibizumab	Lucentis	https://www.accessdata.fda.gov/scripts/cder/daf/index.cfm?event=overview.process&ApplNo=125156
Dasatinib	Sprycel	https://www.accessdata.fda.gov/scripts/cder/daf/index.cfm?event=overview.process&ApplNo=021986
Darunavir ethanolate	Prezista	https://www.accessdata.fda.gov/scripts/cder/daf/index.cfm?event=overview.process&ApplNo=021976
Varenicline tartrate	Chantix	https://www.accessdata.fda.gov/scripts/cder/daf/index.cfm?event=overview.process&ApplNo=021928
Decitabine	Dacogen	https://www.accessdata.fda.gov/scripts/cder/daf/index.cfm?event=overview.process&ApplNo=021790
Alglucosidase alfa	Myozyme	https://www.accessdata.fda.gov/scripts/cder/daf/index.cfm?event=overview.process&ApplNo=125141
Anidulafungin	Eraxis	https://www.accessdata.fda.gov/scripts/cder/daf/index.cfm?event=overview.process&ApplNo=021632
Rotigotine	Neupro	https://www.accessdata.fda.gov/scripts/cder/daf/index.cfm?event=overview.process&ApplNo=021829
Lubiprostone	Amitiza	https://www.accessdata.fda.gov/scripts/cder/daf/index.cfm?event=overview.process&ApplNo=021908
Ranolazine	Ranexa	https://www.accessdata.fda.gov/scripts/cder/daf/index.cfm?event=overview.process&ApplNo=021526
Sunitinib malate	Sutent	https://www.accessdata.fda.gov/scripts/cder/daf/index.cfm?event=overview.process&ApplNo=021938
Silodosin	Rapaflo	https://www.accessdata.fda.gov/scripts/cder/daf/index.cfm?event=overview.process&ApplNo=022206
Conivaptan hydrochloride	Vaprisol	https://www.accessdata.fda.gov/scripts/cder/daf/index.cfm?event=overview.process&ApplNo=021697
Lenalidomide	Revlimid	https://www.accessdata.fda.gov/scripts/cder/daf/index.cfm?event=overview.process&ApplNo=021880
Abatacept	Orencia	https://www.accessdata.fda.gov/scripts/cder/daf/index.cfm?event=overview.process&ApplNo=125118
Sorafenib tosylate	Nexavar	https://www.accessdata.fda.gov/scripts/cder/daf/index.cfm?event=overview.process&ApplNo=021923
Mecasermin rinfabate	Iplex	https://www.accessdata.fda.gov/scripts/cder/daf/index.cfm?event=overview.process&ApplNo=021884
Hyaluronidase (human recombinant)	Hylenex recombinant	https://www.accessdata.fda.gov/scripts/cder/daf/index.cfm?event=overview.process&ApplNo=021859
Deferasirox	Exjade	https://www.accessdata.fda.gov/scripts/cder/daf/index.cfm?event=overview.process&ApplNo=021882
Nelarabine	Arranon	https://www.accessdata.fda.gov/scripts/cder/daf/index.cfm?event=overview.process&ApplNo=021877
Ivabradine hydrochloride	Corlanor	https://www.accessdata.fda.gov/scripts/cder/daf/index.cfm?event=overview.process&ApplNo=206143

© 2025 Kasahara S et al. *JAMA Network Open*.

Posaconazole	Noxafil	https://www.accessdata.fda.gov/scripts/cder/daf/index.cfm?event=overview.process&ApplNo=022003
Inulin		
Mecasermin	Increlex	https://www.accessdata.fda.gov/scripts/cder/daf/index.cfm?event=overview.process&ApplNo=021839
Nepafenac	Nevanac	https://www.accessdata.fda.gov/scripts/cder/daf/index.cfm?event=overview.process&ApplNo=021862
Doripenem	Doribax	https://www.accessdata.fda.gov/scripts/cder/daf/index.cfm?event=overview.process&ApplNo=022106
Ramelteon	Rozerem	https://www.accessdata.fda.gov/scripts/cder/daf/index.cfm?event=overview.process&ApplNo=021782
Tipranavir	Aptivus	https://www.accessdata.fda.gov/scripts/cder/daf/index.cfm?event=overview.process&ApplNo=021814
Tigecycline	Tygacil	https://www.accessdata.fda.gov/scripts/cder/daf/index.cfm?event=overview.process&ApplNo=021821
Galsulfase	Naglazyme	https://www.accessdata.fda.gov/scripts/cder/daf/index.cfm?event=overview.process&ApplNo=125117
Exenatide	Byetta	https://www.accessdata.fda.gov/scripts/cder/daf/index.cfm?event=overview.process&ApplNo=021773
Luliconazole	Luzu	https://www.accessdata.fda.gov/scripts/cder/daf/index.cfm?event=overview.process&ApplNo=204153
Tocilizumab	Actemra	https://www.accessdata.fda.gov/scripts/cder/daf/index.cfm?event=overview.process&ApplNo=125276
Tamibarotene		
Entecavir	Baraclude	https://www.accessdata.fda.gov/scripts/cder/daf/index.cfm?event=overview.process&ApplNo=021797
Pramlintide acetate	Symlin	https://www.accessdata.fda.gov/scripts/cder/daf/index.cfm?event=overview.process&ApplNo=021332
Rasagiline mesylate	Azilect	https://www.accessdata.fda.gov/scripts/cder/daf/index.cfm?event=overview.process&ApplNo=021641
Insulin detemir	Levemir	https://www.accessdata.fda.gov/scripts/cder/daf/index.cfm?event=overview.process&ApplNo=021536
Carglumic acid	Carbaglu	https://www.accessdata.fda.gov/scripts/cder/daf/index.cfm?event=overview.process&ApplNo=022562
Ioflupane I 123	Datscan	https://www.accessdata.fda.gov/scripts/cder/daf/index.cfm?event=overview.process&ApplNo=022454
Deferiprone	Ferriprox	
Repaglinide		
Cysteamine bitartrate		

eReferences

1. Kanehisa M, Furumichi M, Tanabe M, Sato Y, Morishima K. KEGG: New perspectives on genomes, pathways, diseases and drugs. *Nucleic Acids Res*. 2017;45(D1):D353-D361. doi: 10.1093/nar/gkw1092.

© 2025 Kasahara S et al. *JAMA Network Open*.

2. US Food and Drug Administration, US Dept of Health and Human Services. Guidance for industry: Expedited programs for serious conditions—Drugs and biologics; 2014. https://www.fda.gov/downloads/Drugs/Guidances/UCM358301.pdf. Accessed September 30, 2024.

3. Michaeli DT, Michaeli T, Albers S, Boch T, Michaeli JC. Special FDA designations for drug development: Orphan, fast track, accelerated approval, priority review, and breakthrough therapy. *Eur J Health Econ*. 2024;25(6), November:979-997. doi: 10.1007/s10198-023-01639-x.

4. Access to Medicine Foundation. 2022 Access to Medicine Index. https://accesstomedicinefoundation.org/resource/2022-access-to-medicine-index. Accessed September 30, 2024. 16. Top. 20 Companies Include GSK plc, Johnson & Johnson, AstraZeneca plc, Novartis AG, Merck KGaA, Pfizer Inc, Takeda Pharmaceutical Co, Ltd, Sanofi, Bayer AG, Roche Holding AG, Novo Nordisk A/S, Eisai Co, Ltd, Boehringer Ingelheim, Gilead Sciences, Bristol Myers Squibb, Astellas Pharma Inc, Daiichi Sankyo Co, Ltd, Merck & Co, Inc, AbbVie Inc, and Eli Lilly and Co.

© 2025 Kasahara S et al. *JAMA Network Open*.
