# Supplementary material for: Approval Disparities for New Drugs in the US and Japan
Source: JAMA Netw Open. 2025 Jun 10;8(6):e2513640. doi: 10.1001/jamanetworkopen.2025.13640 (PMC12152698; doi:10.1001/jamanetworkopen.2025.13640)
Supplement: Supplement 2. — Data Sharing Statement [file jamanetwopen-e2513640-s002.pdf]

Data Sharing Statement

Kasahara. Approval Disparities for New Drugs in the US and Japan. *JAMA Netw Open*.
Published June 10, 2025. doi:10.1001/jamanetworkopen.2025.13640

Data
Data available: Yes
Data types: Data dictionary
How to access data: shingo.kasahara@keio.jp
When available: With publication

Supporting Documents
Document types: None

Additional Information
Who can access the data: researchers whose proposed use of the data has been approved
Types of analyses: for a specified purpose
Mechanisms of data availability: after approval of a proposal
